# The Potential Roles of Artemisinin and Its Derivatives in the Treatment of Type 2 Diabetes Mellitus

**DOI:** 10.3389/fphar.2020.585487

**Published:** 2020-11-26

**Authors:** Ya-yi Jiang, Jia-cheng Shui, Bo-xun Zhang, Jia-wei Chin, Ren-song Yue

**Affiliations:** ^1^ Hospital of Chengdu University of Traditional Chinese Medicine, Chengdu, China; ^2^ Department of Endocrinology, Guang’anmen Hospital of China, China Academy of Chinese Medical Sciences, Beijing, China

**Keywords:** Artemisinin, artemisinin derivatives, type 2 diabetes, complications, therapy, pharmacology

## Abstract

Type 2 diabetes mellitus (T2DM) is a chronic disease that has become a global public health problem. Studies on T2DM prevention and treatment mostly focus on discovering therapeutic drugs. Artemisinin and its derivatives were originally used as antimalarial treatments. In recent years, the roles of artemisinins in T2DM have attracted much attention. Artemisinin treatments not only attenuate insulin resistance and restore islet ß-cell function in T2DM but also have potential therapeutic effects on diabetic complications, including diabetic kidney disease, cognitive impairment, diabetic retinopathy, and diabetic cardiovascular disease. Many *in vitro* and *in vivo* experiments have confirmed the therapeutic utility of artemisinin and its derivatives on T2DM, but no article has systematically demonstrated the specific role artemisinin plays in the treatment of T2DM. This review summarizes the potential therapeutic effects and mechanism of artemisinin and its derivatives in T2DM and associated complications, providing a reference for subsequent related research.

## Introduction

Diabetes mellitus is a group of metabolic disorders characterized by prolonged elevated blood glucose levels (Punthakee et al., 2018). Currently, the number of patients with diabetes mellitus has reached more than 422 million worldwide, and this figure is predicted to increase to 693 million by 2045, which illustrates the severity of this public health problem (Zimmet et al., 2016; Cho et al., 2018). In general, the majority of diabetes mellitus cases can be divided into type 1 diabetes and type 2 diabetes (T2DM) according to different pathological characteristics, and T2DM cases account for more than 95% of the total diabetes population (Elkhidir et al., 2017). The main pathological basis of T2DM is insulin resistance (IR) and relatively insufficient insulin secretion. However, prolonged hyperglycemia may cause irreversible damage to the function of pancreatic islet ß-cells, resulting in an absolute decrease in insulin secretion (Tan and Cheah, 1990; Johnson and Luciani, 2010). Without good control, hyperglycemia will cause a series of severe complications, including renal failure, heart attack, vision loss, unhealed wounds, cognitive impairment, and increased risk of premature death (Schena and Gesualdo, 2005; Tang et al., 2013; Saedi et al., 2016; Stitt et al., 2016; Jia et al., 2018). Unfortunately, to date, the pathogenesis of T2DM has not been fully elucidated; therefore, symptomatic treatments, such as those that lower blood sugar, are still the main treatments. However, these hypoglycemic agents are neither effective for the overall improvement of patients’ condition nor completely prevent the progression of T2DM. Therefore, it is urgent to find a better drug to treat T2DM.

**FIGURE 1:**
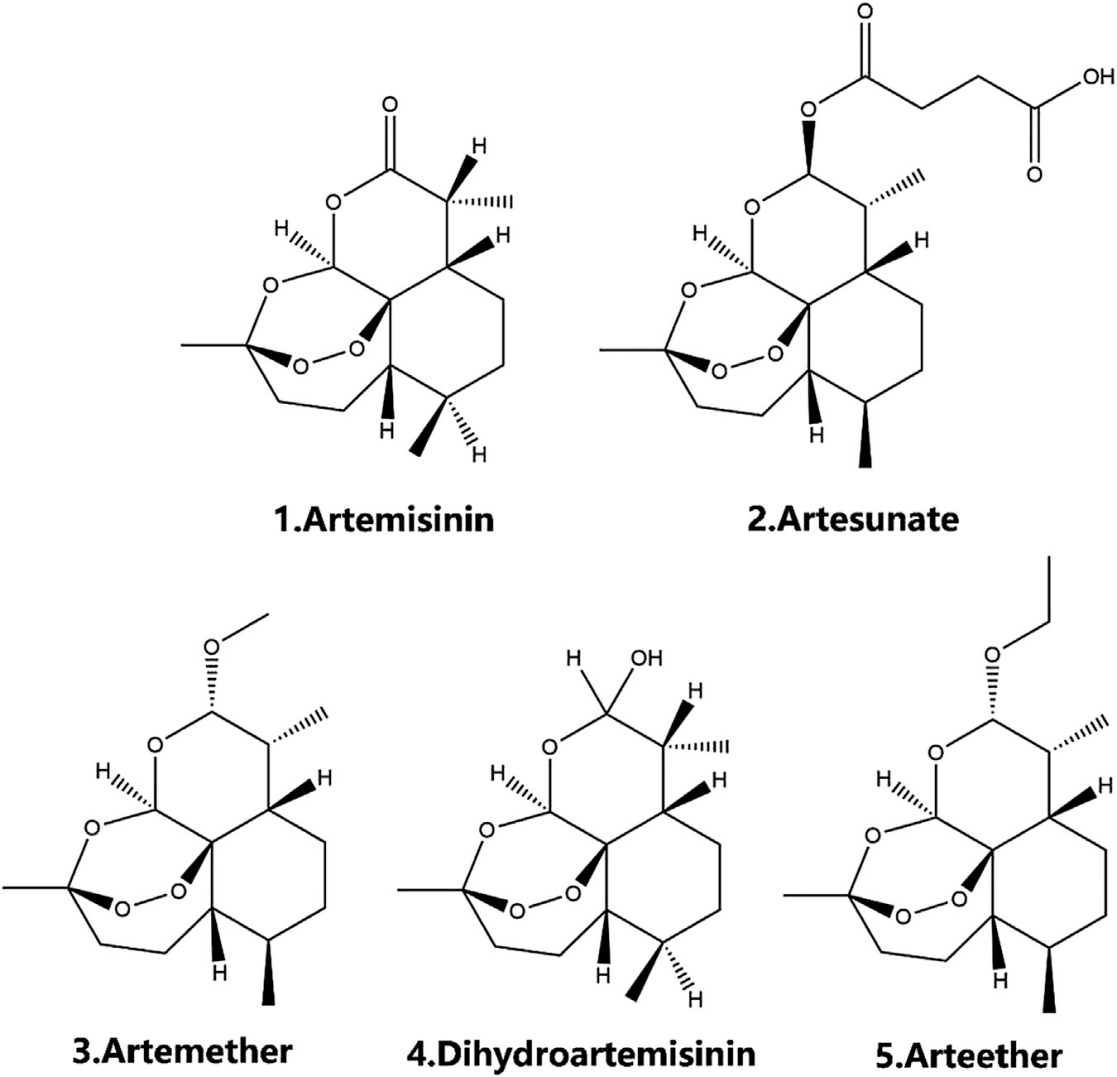
The chemical structure of artemisinin family.

**FIGURE 2:**
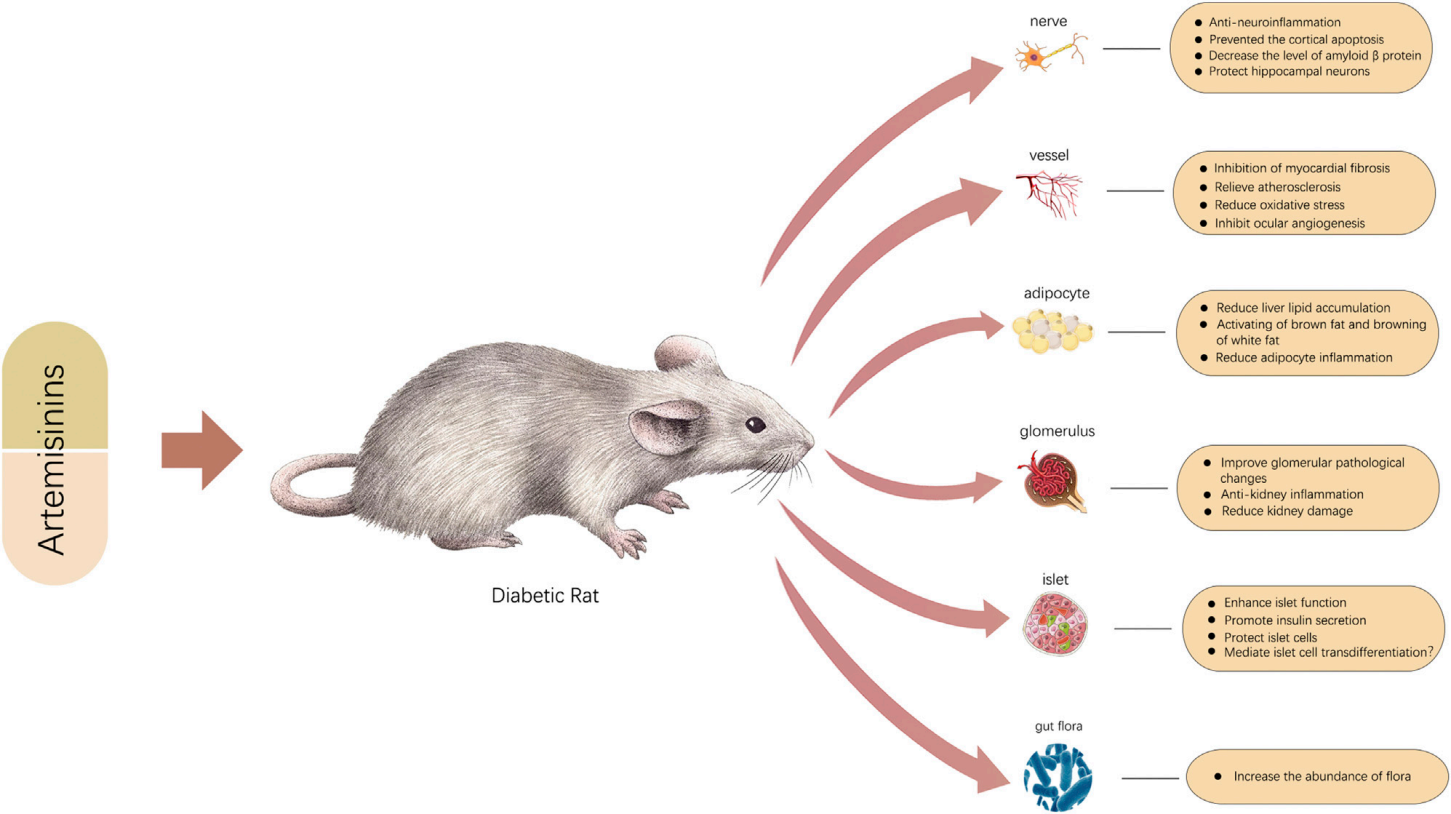
Graphic abstract.

Traditional Chinese medicine (TCM) has been applied in the clinic for thousands of years and has been a substantial frontline treatment for treating various diseases. TCM has received increasing attention since it plays a huge role as a source of new drugs in modern drug discovery. In 2015, Youyou Tu was awarded the Nobel Prize in Physiology or Medicine for her discovery of the natural antimalarial drug artemisinin, which is extracted from *Artemisia annua* (*A. annua*) (Klayman, 1985; Youyou et al., 2015). Since then, semisynthetic derivatives of artemisinin have been gradually developed, including artesunate, artemether, dihydroartemisinin, artelinic acid, and arteether, all of which show great promise in the treatment of malaria (Klayman, 1985; Teja-Isavadharm et al., 2010; Morris et al., 2011; Morris et al., 2013) (Figure 1). As research has progressed, the effects of artemisinin and its derivatives have been greatly extended, and these compounds are widely used in antitumor (Verma et al., 2017; Wang et al., 2017; Wei and Liu, 2017), antifibrosis (Cao et al., 2016; Wang et al., 2019a), immunosuppressive (Li et al., 2013; Hou and Huang, 2016), antivirus (Sharma et al., 2014), antiatherosclerosis (Du et al., 2019; Jiang et al., 2020), antiobesity (Lu et al., 2016), and antidiabetes (Ho et al., 2014) treatments. Recently, increasing evidence has shown that artemisinins have significant therapeutic effects on metabolic diseases, especially diabetes, obesity, and hypercholesterolemia. Although much evidence suggests a positive role for artemisinins in the treatment of T2DM and its complications, no article has systematically demonstrated the specific roles artemisinins have played in the treatment of T2DM. As shown in Tables 1,2 and Figure 2, in this review, we summarize the roles of artemisinin and its derivatives in T2DM, including a variety of diabetes complications, expecting to provide new ideas for future therapy development.

**TABLE 1:** Effects of artemisinin and its derivatives on T2DM.

Disease	Artemisinins	Vivo or vitro	Model	Dosage and duration	Described effects	Potential mechanism	Author
Diabetes	Ethanolic leaf extracts of artemisia annua	In vivo	Wistar rats	100, 200 and 300 mg/kg/day, 28 days	HDL ↓ LDL↑ total protein↑ ALT, AST, albumin and total and direct bilirubin (no alter) bicarbonate ions↑ renal function markers (no alter) glucose concentrations ↓		Ubana (Eteng et al., 2013)
	Artemether	In vivo	Male C57BL6/J mice, HFD	20 mg/kg or 15 μM, biw, 8 weeks	Weight gain↓ adipose tissues↓brown adipose tissue↓ white adipose tissue↓ ITT and GTT (improved)	UCP1↑ p38 MAPK/ATF2 axis↑ the Akt/mTOR pathway ↓	Lu et al. (Lu et al., 2016)
	Artemether dihydroartemisinin	In vitro	C3H10T1/2 cells and 3T3-L1 cells, BMP4 or BMP7	15 μM, 8 days	thermogenesis↑	UCP1↑ PGC1α ↑ PRDM16↑ mitochondrial gene (cyto C) ↑	Lu et al. (Lu et al., 2016)
	Artemisia annua leaf extract	In vivo	C57BL/6J mice, HFD	400 mg/kg/day, 8 w	Body and liver weights↓ blood glucose levels↓ total serum cholesterol↓ ITT and GTT (improved) TG↓	α-glucosidase↑phosphorylated ACC ↑ COX-2 ↓ChREBP and SREBP1 expression↓	Kim et al. (Kim et al., 2016)
	Artesunate	In vitro	3T3-L1 preadipocytes, Human primary preadipocytes	5 μM, 8 days，5 μM, 20 days	Lipid accumulation↓ TG↓ perilipin A↓ leptin↓ adiponectin (not alter) number of human adipocytes (no alter)	C/EBP-α↓ppar-γ↓STAT-3↓FAS↓	Jang et al. (Jang 2016)
	Artemisinic acid	In vitro	Human adipose tissue-derived mesenchymal stem cells	0, 10, 50, 100, 200, 400 μM, 3 days	Lipid accumulation↓ TG↓ adipocyte protein↓	C/EBP-α↓C/EBP-δ↓PPAR γ↓fatty acid translocase (CD36)↓liver X receptor↓JNK↓GLUT4↓VEGF↓IL-6↓GPDH↓LXR α↓LPL↓aP2↓NF-kB (no alter) AP-1↓p42/44 MAPK and p38 MAPK (no alter) MMP-2↓	Lee et al. (Lee et al., 2012a)
	Artemether	In vivo	db/db mice	200 mg/kg/day, 2 w	Food intake↓ weight increase rate↓ AUCs of GTT↓ fasting blood glucose↓ hyperinsulinemia↓ HOME-IR↓ insulin sensitivity↑ islet vacuolar degeneration↓ islet morphologies↑ islet cell numbers and size↑ hepatic steatosis↓ apoptosis of pancreatic ß cells↓ insulin secretion↑		Guo et al. (Guo et al., 2018)
	Artemether	In vitro	αTC1 cells	10 μM, 72 h	proglucagon↓ glucagon↓ processed glucagon peptides↓ Insulin↑ excitability↑Ca^2+^ levels↓	αTC1 cells: ARX↓ Pax4 and Mnx1↑PI3P ↓phosphorylated Akt↓ PI3K ↓gephyrin↑ mRNA gephyrin (no alter) gephyrin enzymatic activity↑Moco↑ mTOR signaling ↓ GABA Signaling↑GABAA-receptor complex↑genes P2rx3, Vamp1, and Nrxn3↑	Li et al. (Li et al., 2017)
	Dihydroartemisinin	In vitro	αTC1 cells	10 μM, 72 h	Induces insulin expression in α cells		
	Arteether artesunate						
	Artemether	In vivo	SD rats, STZ	20 mg/kg/day, 200 mg/kg/day, 7 days or 16 days	Fasting glucose levels↓ baseline blood glucose levels↓ GTT (improved) AUCs of GTT↓		Li et al. (Li et al., 2017)
	Artesunate	In vivo	Wild-type mice	1 mg/ml, 3 months	Pancreatic islet size↑	Arx ↓	
	Artemether	In vitro	Islets isolated from lineage-tracing mice	10 μM, 24 h	Insulin expression in α cells↑ insulin-positive cells↑ islet mass↑ blood glucose levels↓		
	Artemether	In vitro	Zebrafish larvae	10 μM, 72 h	Islet morphology (improved) α cell numbers↓β cell number↑ glucose↑→↓		
	Artemether	In vitro	Human islets	10 μM, 72 h	Insulin (no alter in low-glucose conditions) insulin↑ (only in high-glucose conditions )	ARX↓ gephyrin↑ GABA-receptor subunit protein levels↑GABA related genes GABRB3 and GABRG2↑α cell-specific genes:EIF4A1, CRYBA2, PDK4, and MUC13↓β cell-specific genes:GNAS and ABCC8↑	
	Artemisinin	In vivo	Male SD rats, STZ	300 mg/kg/d, 4 w	glomerular hypertrophy↓glomerular capillary dilatation↓hyperplasia of glomeruli↓FBG↓ urine protein↓ BUN↓ Cr↓LDL cholesterol↓TG↓TC↓insulin↑	After artemisinin treatment:down-regulated genes: Id1, Cdkn1a, Hmgcs2 and Rarres2 GO term: (1) biological process:organic anion transport (2) cellular component:extracellular region part (3) molecular function:organic anion transmembrane transporter activity up-regulated genes:Igfbp1, sult1a1, pigr and Steap4 GO term: (1) biological process:response to hormone (2) cellular component:extracellular space (3) molecular function:protein binding KEGG pathways:“Glycine, serine and threonine metabolism,” “complement and coagulation cascades,” “p53 signaling pathway,” “TGF-β signaling pathway,” and “PPAR signaling pathway”	Xiang et al. (Xiang et al., 2019)
	Artesunate	In vitro	INS-1 cell, IL-1β	5 μM–50 μM, 2 h	Insulin secretion↑ (no alter if without IL-1β) viability of INS-1 cells↑apoptosis of pancreatic ß-cells↓	NF-κB↓ (no alter if without IL-1β) p65 proteins↓ iNOS ↓ NO↓SIRT1↑	Yu et al. (Yu et al., 2016)
	Artesunate	In vivo	Female NOD mice	1 mg/ml	β-cell mass↑α-cell mass (no alter) β-cell functional maturity↑insulitis↓	The frequency of IFN-γ–producing T cells↓IL-4–producing T cells↑Th1 cells ↓ Th2 cells↑TNF-α↓IL-6↓ Ins1 and Ins2 ↑MafA↑ Ucn3↑NeuroD1↑Hnf1b Sox9 Pdx-1 (no alter) Ngn3↓	Li et al. (Li et al., 2019d)
	Artemisinin dihydroartemisinin	In vitro	INS-1and MIN6，palmitate	0, 200, 500, 1,000, 2000, 4,000, 8,000, 16,000 nM, 12 h or 24 h	viability↓ (with increasing concentration) cytotoxicity:artemisinin ＜ dihydroartemisinin significant damage (no alter) drug toxicity (little)	INS-1 apoptosis and ER stress related mRNA (GRP78, CHOP, PDI) ↑ (at 1,000 and 2000 nM) INS-1 apoptosis and mRNA (GRP78, CHOP, PDI) (no alter) (100, 200 nM)	Chen et al. (Chen et al., 2020)
	Artemether	In vitro	Male islets from ins1-h2b-mcherry×Gcg-cre×rosa26-stop-yfp triple transgenic mice	10 μm, 72 h	Insulin secretion↓	Arx↓α to ß cell transdifferentiation (−) ß cell identity↓ Ins1↓ Ins2↓ ß cell markers (Ucn3, Mafa, Pdx1, and Slc2a2)↓apoptosis of ß cell (no alter) δ cell markers (Sst and Hhex)↓β cell glucose uptake↓Slc2a2↓glucose transporter (Glut2)↓calcium response of ß cells↓ expression of the glucagon receptor and GABA receptors (no detect)	Van der Meulen.et al. (van der Meulen et al., 2018)
	Artemether	In vitro	Human donor islets	10 µm, 72 h		INS expression (no alter)	
	Artemether	In vitro	C57BL/6 wild-type mouse islets	10 µm, 72 h		α cell genes↓ (Gcg, Mafb, Irx1)	
	Artesunate	In vivo	Glucagon-CreERT2; Rosa-LSL-eYFP male mice	1 mg/ml, 3 months	GTT (improved) fasting and 2 h ending plasma glucose levels (no alter) body weight↓ glucose tolerance (no alter) islet number (no alter) islet area (no alter)	α-To-β cell transdifferentiation (—) number of YFP + cells (no alter) ß cell area (no alter) islet size distribution (no alter)	Ackermann et al. (Ackermann et al., 2018)
	Artemether	In vitro	Mouse pancreatic islet	10 μM, 72 h	Insulin expression↑	In α cells: (1) ß cell-specific genes Ins1, igf1r, Pdx1, Nkx6-1, Nkx2-2, iapp, Foxo1, Abcc8, and slc2a2 ↑ “regulation of gene expression in ß cells”↑ “β cell development”-specific gene sets↑ in ß cells: Genes in the insulin secretion, glucagon signaling, and FoxO signaling pathways such as Ucn3, Nkx6-1 Pcsk2, and FoxO1↓ key ß cell genes:INS1/2, SLC2A2, ISL1, GCGR, UCN3, and SCG5↓SPP1↓ Igf1R↓	(Marquina-Sanchez et al., 2020)
	Artemether	In vitro	Human pancreatic islet	10 μM, 72 h	Insulin-expressing↑	In α cells: (1) β cell-specific genes IAPP, DLK1, ABCC8, PDX1, MAFA, NKX6-1, and NKX2-2↑ (2) α cell-specific genes GCG, ARX, and TTR↓ “insulin secretion”pathways↑ “insulin signaling”pathways↑ insulin/glucagon double positive cells↑ in ß cells: ß cells↑key ß cell genes:INS1/2, SLC2A2, ISL1, GCGR, UCN3, and SCG5 (no alter) SPP1↑ lgf1r↑	
	Artemether	In vivo	Male db/db mice	100 mg/kg, 200 mg/kg, 4 w	Rates of weight gain↓food and water intake↓ fasting blood glucose levels↓GTT and ITT (improved) islet function, insulin resistance and insulin sensitivity (improved) serum lipid levels (TC, TG and FFA)↓ islet vacuolar degeneration and hepatic steatosis (improved)	AMPK↑ GLUT-4↑ IRβ↑ expression of inflammatory factors↓	Fu et al. (Fu et al., 2020)

**TABLE 2:** Effects of Artemisinins on T2DM Associated Complications.

Disease	Artemisinins	Vivo or vitro	Model	Dosage and duration	Described effects	Potential mechanism	Author
Diabetic kidney disease	Artemether	In vivo	Male db/db mice	0.67 g/kg, 12 w	Urinary albumin excretion↓ diabetic kidney hypertrophy↓kidney weight↓ GBM and TBM thickening↓ FPW↓ diabetic symptoms (improved) hyperglycemia↓ fasting blood glucose levels↓ body weight↓ HbA1c↓ urinary glucose levels↓ serum insulin↑ the islet area/pancreas area and islet density values↑ insulin, glucagon and somatostatin content in islets (improved)	RER↑ energy expenditure (no alter) renal mitochondrial H_2_O_2_ production↓ serum and urine H_2_O_2_ level↓ renal cortical PGC-1α↑ mitochondrial MPC content↑catalase and SOD2 expression (no alter) PDH and PDK1 expression (no alter) PDP1↑MPC1 and MPC2↑	Han et al. (Han et al., 2019)
	Artesunate	In vitro	HBZY-1 cells, HG	15, 30 μg/ml, 24 h	High glucose-induced proliferation of HBZY-1 cells↓	Inflammatory responses↓IL-6, IL-1β, and TNF-α↓ oxidative stress↓ROS ↓MDA↓ SOD activity↓ expression of ECM proteins↓laminin, collagen IV, fibronectin↓TLR4, MyD88↓, NF-κB p65, NF-κB p-p65, and NLRP3↓	Sun et al. (Sun et al., 2018)
	Artemisinin	In vivo	Male SD rats, STZ	25, 50, 75 mg/kg, 8 w	Kidney indexes↓weights↑ blood glucose↓24 h UAER, BUN and Scr↓ mesangial cells proliferation↓ thickness of glomerular capillary basement membrane↓ ECM↓	T-SOD ↓GSH-Px↓ MDA↓ Nrf2 signaling pathway↑TGF-β1 protein expression↓ antioxidant proteins HO-1 and NQO1 and Nrf2-related proteins↑	Zhang et al. (Zhang et al., 2020)
	Artemisinin	In vivo	Male SD rats, STZ	300 mg/kg/d, 3 w, 6 w	Kidney/body weight ratio↓ Ccr↓ UAER↓ extensive kidney lesion↓ glomerular mesangial cell proliferation↓ mesangial area broadening↓ thickening of basement membrane↓ glomerular visceral epithelial cell hypertrophy↓ mesangial matrix↓	PDGF-B↓PKC activity protein expressions of fibronectin↓ collagen IV ↓ TIMP-2↓ MMP-2 ↑	Zhang et al. (Zhang et al., 2014a; Zhang et al., 2014b; Zhang et al., 2014c)
	Artemisinin	In vivo	Male SD rats, STZ	300 mg/kg/day, 4 w	Glomerular mesangial cell proliferation↓ mesangial area broadening↓ thickening of basement membrane↓	DNA-binding activity of AP-1↓ c-fos and c-jun mRNA and protein↓ NF － κB ↓	Zhou et al. (Zhou et al., 2014a; Zhou et al., 2014b; Zhou et al., 2014c)
	Artemether	In vivo	Male C57BL/6J mice, STZ	0.67 g/kg, 8 w	Serum insulin↑mean islet area values (no alter) glucagon/insulin↓somatostatin/insulin↓polydipsia↓polyuria↓polyphagia↓increased feces production↓ fasting blood glucose↓HbA1c↓urinary glucose levels↓ urinary excretion of albumin↓serum TP and albumin levels↑kidney weight↓mesangial matrix areas↓hypertrophic glomeruli ↓proximal tubules↓ tubular injury biomarkers (NAG, NGAL, Kim-1)↓	Mitochondrial function (improved) mitochondrial H_2_O_2_ release rate↑ PDK1↓ PGC-1α↑catalase↑ SOD2 (no alter)	Wang et al. (Wang et al., 2019b)
	Artesunate	In vivo	Male SD rats, STZ	30 mg/(kg·d), 10 mg/(kg·d)	Urine protein↓Cr↓BUN↓	TLR4↓ IL-8↓	Nie et al. (Nie et al., 2015)
Diabetic retinopathy	Artesunate	In vitro	White rabbits, VEGF165 + Bfgf	Single intravitreal injection of (20, and 40 µg/mL)	Retinal neovascularization↓ vascular dilatation, tortuosity↓iris neovascularization↓fluorescein leakage into the anterior chamber↓ (within 1 week) retinal vascular disorders were completely blocked (6 months) retinal edema↓epiretinal fibrovascular membranes↓ (6 months)	VEGFR2↓PKCα ↓PDGFR↓ (1 month)	Zong et al. (Zong et al., 2016)
	Artesunate	In vitro	Huvec, HG	10 µg/ml, 20 µg/ml, 40 µg/ml		ICAM-1↓MMP-9↓	Ge et al. (Ge et al., 2019)
	Artesunate	In vitro	On male cynomolgus monkeys, VEGF165 + bFGF	Single intravitreal injection of (20 μg)	Fluorescein leakage↓ (within 1 week) macular edema↓macular retinal tear, an epiretinal membrane, and retinal detachment (improved) RNFL thickness or C/D (no alter) iris neovascularization (vanished within 3 days) fluorescein leakage into the anterior chamber (vanished within 2 weeks) corneal neovascularization in a case of severe retinal detachment (improved after 3 months treatment)		
	Dihydroartemisinin	In vitro	HUVECs	25 μM, 100 μM, 12 h or 24 h	Endothelial cell proliferation and migration↓	VEGFR2↓NF-κB signaling↓ cytosolic retention (+) nuclear p65 staining (−) NF-kB translocation (blocked) IκB-α protein↑activity of VEGFR2 promoter↓ binding of p65 protein↓	Dong et al. (Dong et al., 2014)
	Dihydroartemisinin	In vitro	HUVECs	20 μm, 24 h	Huvec proliferation↓ cell density↓	PCNA↓ induction of apoptosis (−) ERK1/2 expression↓ total ERK1/2/β-actin↓ phospho-ERK1/2/total-ERK1/2↓ ERK1 (MAPK3) expression↓ ERK2 (MAPK1) expression↓ c-fos and c-Myc↓	Dong et al. (Dong et al., 2015)
	Artemisinin	In vitro	Human retinal pigment epithelial cell line, H_2_O_2_ for 24 h	30 μM, 2 h	Cell death (attenuated) LDH leakage↓Nuclei condensation↓	Mitochondrial membrane potential↑caspase 3/7↓ROS↓phosphorylation intensity of ERK1/2 ↑ phosphorylation of CREB (Ser133)↑	Chong et al. (Chong and Zheng 2016)
	Artesunate	In vivo	5 patients (corneal and iris neovascularization, which were secondary to fundus ischemic diseases and retinal neovascularization)	1 or 2 (20 μg/ml, 4 ml) injections	Retinal neovascularization↓ papilledema↓ IOP↓		Li et al. (Li et al., 2019a)
	Artemisinin	In vitro	Retinal neuronal RGC-5 cells, H_2_O_2_	Intravitreous injection (6.25–100 µM)	Viability of cells↑	Oxidative damage↓ mitochondrial membrane potential↑ apoptosis ↓Bax↓cellular ROS levels ↓phosphorylation of p38 and ERK1/2↑ phosphorylation of JNK1/2 and mTOR (no alter)	Yan et al. (Yan et al., 2017)
	Artemisinin	In vitro	Human retinal pigment epithelial cell line (D407), H_2_O_2_	(3.125 μM–100 μΜ) 2 h	apoptosis↓LDH ↓	Mitochondrial membrane potential↑ROS generation ↓activation of AMPK↑	Li et al. (Li et al., 2019b)
	Artesunate	In vitro	HUVECs	(0, 25, or 100 μM) 12 h or 24 h	proliferation↓ apoptosis↑	Mitochondrial Bax↑mitochondrial Bcl-2↓cytochrome C release↑ mitochondrial membrane potential↓cleavage of caspase 3 and 9↑Fas and fas ligand (no alter) caspase 8 cleavage (no alter) p38 MAPK activation↑ROS↑ (iron-dependent) (in a concentration-dependent) VEGF2↓	Cheng et al. (Cheng et al., 2013)
	Artesunate	In vitro	HDMECs	(0, 25, or 100 μM) 12 h or 24 h	proliferation↓	Cleavage of caspase 3 and 9↑Fas and fas ligand (no alter) caspase 8 cleavage (no alter)	
Diabetic cardiovascular disease	Artemisinin	In vivo	Male Wistar rats, STZ	75 mg/kg, 4 w	Polydipsia↓ polyphagia↓ polyuria↓ weight↑ (compare to diabetes group) plasma glucose (no significantly different with normal group) left ventricular end-systolic dimension↓ left ventricular end-diastolic dimension↓ left ventricular ejection fraction↑ fractional shortening↑ E/A↑ necrosis↓	Collagen fibers↓TGFβ-1↓CollagenⅠ↓CollagenⅢ↓TNF-α↓NF-KB↓IL-1β (no alter)	Li et al. (Li et al., 2016)
	Artesunate	In vitro	Rabbits	25 mg/kg	Plasma cholesterol↓ triglyceride↓ liver steatosis (improved) area of aortic root lesions↓	LDL↓ KLF-2 protein↑ apoA-I mRNA↑	Wang et al. (Wang et al., 2013)
	Artemisinin	In vitro	Human monocytic THP-1 cells, phorbol myristate acetate	20–80 μg/ml, 4 h		Extracellular matrix metalloproteinase inducer and MMP-9↓PKCδ⁄JNK⁄p38⁄ERK MAPK phosphoryla- tion (blocked) MMP-9 activity↓	Wang et al. (Wang et al., 2011a)
	Artemisinin	In vitro	Human monocytic THP-1 cells, phorbol myristate acetate	0–160 μg/ml, 4 h	Monocytes proliferation↑	mRNA levels and secretion of TNF-α↓IL-1ß↓IL-6↓ (80 μg/ml almost completely inhibited ) phosphorylation of IKKα/ß↓the phosphorylation and degradation of IκBα↓the nuclear translocation of the NF-κB p65 subunit↓	Wang et al. (Wang et al., 2011b)
	Artemisinin	In vitro	HUVECs, TNF-α	0–300 µM, 4 h	Viability of the HUVECs was > 90% (<200 µM) monocyte adhesion to HUVECs↓	ICAM-1↓VCAM-1↓ activation of the NF-κB and MAPK signaling pathways↓ phosphorylated ERK1/2, p38 and JNK↓blocks NF-κB translocation↓	Wang et al. (Wang et al., 2016b)
	Artemisinin	In vitro	VSMC, PDGF-BB	10 and 30 μM, 1 h	PDGF-BB-induced proliferation↓	ROS↓ phosphorylation of ERK1/2↓ MMP9 expression↓scratch wound healing↓migration ↓	Lee et al . (Lee et al., 2014)
	Artemisinin	In vivo	Male ApoE–/–mice	50 and 100 mg/kg	Vascular smooth muscle cell hyperplasia and fibrosis↓ lesion size↓ serum TC and TG levels ↓ LDL cholesterol and HDL cholesterol contents (no alter)	NLRP3 inflammasome activation↓ IL-1β ↓ IL-18↓ VCAM-1 and ICAM-1 expression↓AMPK phosphorylation↑	Jiang et al. (Jiang et al., 2020)
	Artemisinin	In vitro	oxLDL-treated macrophages	100 mg/ml	Foamy transformation and inflammatory response↓	IL-1β ↓ IL-18↓ AMPK phosphorylation↑ p-NF-κB levels↓ ASC and cleaved caspase-1↓AMPK/NF-κB/NLRP3 inflammasomes signaling ↓	
	Artesunate	In vivo	Male ApoE −/− mice	1.5, 5 and 15 mg/kg/day, 6 months	Progression of atherosclerosis lesion formation↓ areas of enface aorta ↓ food uptake, body weight and plasma lipids level (no alter)	TNF-α and IL-6↓IL-8 and MCP-1 mRNA and protein↓	Jiang et al. (Jiang et al., 2016)
	Artesunate	In vitro	HUVECs	2.5–100 μg/ml, 24 h		MCP-1 and IL-8 (treated with TNF-α no alter) (treated with LPS ↓)	
	Artemisinin	In vivo and in vitro	Male ApoE −/− mice	50 mg/kg/day, 100 mg/kg/day, 8 weeks	Plaque area↓	Expression of contractile phenotypic markers (αSMA, SM22 α, calponin1, and SMMHC) ↑vascular smooth muscle contraction functional set was the most significantly changed pathway	Du et al. (Du et al., 2019)
	Artemisinin	In vitro	MOVAS, PDGF-BB	50 µM or 100 μm, 2 h	PDGF-activated MOVAS migration, proliferation↓	Level of proteins involved in the contractile phenotype↑αSMA, SM22 α, calponin1 and SMMHC↑	
	Artemisinin	In vitro	VSMCs, TNF-α	100 Mm, 2 h	Proliferation, migration of VSMCs↓	Expression of PCNA↓ MMP-2↓ MMP-9↓NO↓ PGE2↓ ROS↓ cyclooxygenase↓ NF-κB pathway subunit NF-κB p65↓phosphorylation of IκB α↓	Cao et al. (Cao et al., 2015)
	Artemisinin	In vivo	ApoE −/− mice, HFD	50, 100 mg/kg/d, 8 w	Foamy macrophage transformation↓atherosclerotic plaque formation↓ LDL-cholesterol and HDL-cholesterol (no alter)	MCP-1, IFN-γ, IL-6 and TNF-α↓AMPK activation↑ macrophage autophagy↑	Cao et al. (Cao et al., 2020)
	Artemisinin	In vitro	Mouse macrophage cell line, oxLDL	100 µM, 24 h		MCP-1, IFN-γ, IL-6 TNF-α ↓AMPK activation↑LC-3II accumulation ↑ P62 degradation↑ (increased autophagyic markers) mTOR and ULK1 phosphorylation↓	
Diabetic cognitive impairment	Artemisia judaica extract	In vivo	Male rats, STZ + HFD	300 mg/kg/day, 21 days	Blood glucose level↓ food and water intakes↓ vacuolation degeneration in the neuronal cells, apoptosis, and infiltration of inflammatory cells (improved)	Antioxidant capacity (improved) activity and mRNA expression of the antioxidant proteins (glutathione peroxidase, glutathione reductase, SOD, SOD2 and catalase)↑GSH content↑ MDA↓ NO↓ brain-derived neurotrophic factor, dopamine, and norepinephrine↑ TNF-α, iNOS, and Bax↓Bcl2↑	Albasher et al. (Albasher et al., 2020)
	Artesunate	In vitro	BV2 mouse microglia cell line, LPS/IFN-γ	1 , 2 and 4 μM, 24 h		PGE2 production↓COX-2 protein expression↓mPGES-1↓TNFα and IL-6 production↓NF-κB and p38 MAPK signaling pathways, NF-κB-driven luciferase expression↓IκB↓IKKα/β phosphorylation↓p38 MAPK↓MAPKAPK2↓	Okorj et al. (Okorji and Olajide 2014)
	Artemether	In vitro	BV2 mouse microglia cell line, LPS	(5–40 μM), 24 h	Viability of BV2 microglia (no alter)	Suppressed pro-inflammatory mediators↓ (NO/iNOS, PGE2/COX-2/mPGES-1 TNFα and IL-6↓) nuclear transactivation of NF-κB↓p38 MAPK Signaling↓ Aβ and BACE-1↓ Nrf2↑ HO-1, NQO1 and GSH↑ ROS ↓	Okorj et al. (Okorji et al., 2016)
	Artesunate	In vivo	Male Wistar rats, thioacetamide	50 or 100 mg/kg/day	Spatial learning ability↑ escape latency↑ working errors ↓		Wu et al. (Wu et al., 2016)
	Artesunate	In vitro	SHSY5Y and CGNs, ammonia	50 or 100 Mm	Cell viability↑	ROS↓ GSH content ↑	
	Artesunate	In vitro	C6 astroglial cells. Ammonia	50 or 100 mM		Glutamate uptake/release activity↑ GS activity↑ intracellular GSH content↑ Na + K + -ATPase activity↑	
	Artemisinin	In vivo	APPswe/PS1dE9 transgenic mice	40 mg/kg/day, 30 days	Neuritic plaque burden↓	Inhibited NF-κB activity ↓NALP3 inflammasome activation↓	Shi et al. (Shi et al., 2013)
	Artemisinin	In vivo	SH-SY5Y and hippocampal neurons, H_2_O_2_	12.5 µM, 2 h	Cell viability (improved) apoptosis↓	Caspase-3↓ROS↓mitochondrial membrane potential loss↓ phosphorylation of AMPK↑	Zhao et al. (Zhao et al., 2019)
	Artemisinin	In vivo and in vitro	SD rats, isoflurane	50, 100 or 200 mg/kg B.wt/day, 18 days	Movement time and distance↑cognition and memory (improved) spatial learning and memory (improved) working memory and object recognition (improved) neuronal apoptosis ↓	Cleaved caspase-3, Bax and Bad↓anti-apoptotic proteins (Bcl-2, Bcl-xL, c-IAP-1, c-IAP-2, xIAP and survivin)↑ acetylation of H3K9 and H4K12↑histone deacetlyases (HDACs) – HDAC-2 and HDAC-3↓JNK signallin↓c-Jun↓ ERK1/2 expression↑	Xu et al. (Xu et al., 2017a)
	Artemisinin	In vitro	PC12 cells, Aβ25–35 or Aβ1–42	25 μM, 1 h	cytotoxicity↓apoptosis↓rescue PC12 cells	LDH↓ROS↓mitochondrial membrane potential loss↓activity of apoptotic caspase ↓caspase 3/7 ↓ERK1/2 ↑	Zeng et al. (Zeng et al., 2017)
	Artesunate	In vivo	BV-2 microglial cells, LPS	0, 0.5, 1.5, 3.5 μM, 18 h	Cytotoxicity (no ≤ 7.5 μM) cell viability (no alter)	NO↓iNOS↓IL-1β↓TLR4↓MyD88↓NF-κB↓	Wang et al. (Wang et al., 2015)

↓indicates inhibition/reduction while↑indicates increase/promotion; GTT, glucose tolerance test; ITT, insulin tolerance test; HDL, high density lipoprotein; LDL, low density lipoprotein; ALT, alanine aminotransfeaseALT; AST, aspartate transaminase; UCP1, uncoupling protein 1; MAPK, mitogen-activated protein kinase; AKT, protein kinase B; ATF2, activating transcription factor-2; mTOR, mammalian target of rapamycin; PGC-1α, peroxisome proliferator-activated receptor γ coactivator 1α; Cyto C, cytochrome C; PRDM16, positive regulatory domain containing 16; TG, triglyceride; ACC, acetyl-CoA carboxylase; ChREBP, carbohydrate-responsive element-binding protein; SREBP1, sterol regulatoryelement-binding protein1; COX-2, cyclooxygenase; FAS, fatty acid synthase; STAT-3/5, signal transducer and activator of transcription-3/5; C/EBPs, CCAAT/enhancer-binding proteins; PPARs, peroxisome proliferator-activated receptors; JNK, Jun N-terminal kinase; GLUT4, glucose transporter-4; VEGF, vascular endothelial growth factor; IL-6, interleukin-6; LPL, lipoprotein lipase; GPDH, glyceraldehyde-3-phosphate dehydrogenase; LXR α, liver X receptor α; MMP, matrix metalloproteinase; AP-1, activator protein 1; AUCs, the areas under the curves; aP2, fatty acid binding proteins; PI3P, phosphatidylinositol 3-phosphate; PI3K, phosphatidylinositol 3-kinase; Moco, molybdenum cofactor; GABA, gamma-aminobutyric acid; BUN, blood urea nitrogen; Cr, creatinine; TC, serum cholesterol; Igfbp1, insulin-like growth factor binding protein 1 gene; iNOS, inducible nitric oxide synthase; NO, nitrite; SIRT1, Sirtuin 1; NF-κB, nuclear factor kappa-B; IκB, inhibitor of kappa B; IFN-γ, interferon γ; ER, endoplasmic reticulum; GLT-4, glucose transporter 4 protein; IRβ, insulin receptorβprotein; AMPK, AMP activated protein kinase; GBM, glomerular basement membrane; TBM, tubular basement membrane; FPW, foot process width; RER, respiratory exchange ratio; HbA1c, glycosylated hemoglobin A1c; SOD, superoxide dismutase; MPC, mitochondrial pyruvate carrier; PDK1, pyruvate dehydrogenase kinase 1; PDH, pyruvate dehydrogenase; PDP1, pyruvate dehyrogenase phosphatase 1; TLR4, toll-like receptor 4; NLRP3, nod-like receptor protein 3; TNF-α, Tumor necrosis factor α; ROS, Reactive oxygen species; MDA, malondialdehyde; ECM, extracellular matrix; MyD88, myeloid differentiation primary response gene 88; GSH, glutathione; TGF-β1, transforming growth factor beta 1; NQO1, NADPH quinone acceptor oxidoreductase 1; Nrf2, nuclear factor-erythroid 2-related factor 2; PDGF, platelet-derived growth factor; HO-1, heme oxygenase-1; TIMPs, tissue inhibitor of metalloproteinases; PKC, protein kinase C; UAER, urinary albumin excretion rate; ICAM-1, intercellular adhesion molecule-1; RNFL, retinal nerve fiber layer; C/D, cup/disc ratio; PCNA, proliferating cell nuclear antigen; ERK, extracellular signal-regulated kinase; LDH, lactate dehydrogenase; CREB, cAMP-response element binding protein; intraocular pressure (IOP); HUVECs, human umbilical vein endothelial cells; Aβ, amyloid-β; MCP-1, monocyte chemoattractant protein-1; LPS, lipopolysaccharide; MOVAS, rat vascular smooth muscle cells; VSMCs, rat vascular smooth muscle cells; ULK1, uncoordinated-51-like kinases 1; PGE2, prostaglandin E2; mPGES-1, microsomal prostaglandin E synthase-1; BACE-1, beta-site amyloid precursor protein cleaving enzyme 1; MAPKAPK2, MAP kinase-activated protein kinase 2.

## Overview of Artemisinin and Its Derivatives

The properties of artemisinins determine their roles. Artemisinin is insoluble in oil and water and is the starting material of semisynthetic derivatives such as artemether, dihydroartemisinin, artesunate, and arteether (O’Neill and Posner, 2004; Gautam et al., 2009). These compounds are characterized by a short half-life, fast onset of effects, and low oral bioavailability (19%–35%) (Thomas et al., 1992; Navaratnam et al., 2000). The artemisinin compounds mentioned above are lipophilic, with the exception of artesunate, which is the only artemisinin derivative for which an intravenous formulation is available; dihydroartemisinin, artemether and arteether are currently administered intramuscularly in various oil formulations (Navaratnam et al., 2000; Krishna et al., 2001). Compared with other artemisinin derivatives, intramuscular artemether and arteether have a longer half-life, which may be attributed to the “depot” effect and/or the local blood supply and the slow and prolonged absorption of the sesame oil preparations at the site of injection (Ashton et al., 1998; Li et al., 1999; Visser et al., 2014). After either parenteral or gastrointestinal administration, artemisinin derivatives are mainly converted into dihydroartemisinin, a bioactive metabolite. The conversion rate of artemether is the lowest (3.7–12.4%), while that of water-soluble artesunate is the highest (25.3–72.7%) (Maggs et al., 1997; Li and Weina, 2010). The transformation of artemisinin and its derivatives into the primary metabolite dihydroartemisinin mostly depends on the action of the liver cytochrome P450 isozyme family, except for artesunate, which depends on the action of common esterases (de Waziers et al., 1990; van Agtmael et al., 1998; van Agtmael et al., 1999; White et al., 1999). Different artemisinin derivatives have different distribution characteristics (Niu et al., 1985). Artemisinin can cross the blood-brain and blood-placenta barriers after intravenous administration (Niu et al., 1985). Similarly, artemether has the ability to cross the blood-brain barrier, and the highest concentration of artemether is in the brain, followed by the heart, lung, skeletal muscles, liver, and kidney after intravenous administration (Jiang et al., 1989; Maggs et al., 1997; Maggs et al., 2000). For artesunate, the highest concentrations were found in rat intestine 10 min after intravenous administration, followed by the brain, liver, kidney, testicle, muscle, fat, heart, serum, eyeball, spleen, and lung in decreasing order (Zhao and Song, 1989). Arteether is the only derivative that cannot directly pass the blood-brain barrier. Twenty-four hours after intramuscular administration, the highest concentration is found in the intestinal tract, followed by the liver, kidneys, spleen, brown fat and brain, and concentrations in the heart, testes, muscle and residual carcass are very low (Navaratnam et al., 2000) (Table 3). In addition, sex (Ashton et al., 1999), diet (Dien et al., 1997; White et al., 1999) and disease state (Batty et al., 1998) have been recognized to contribute significantly to the metabolism of artemisinin and its semisynthetic derivatives. Details on the pharmacokinetics of artemisinin and its derivatives have been described in the relevant literature and will not be described here.

**TABLE 3:** The properties of artemisinin and its derivatives.

Artemisinin family	Solubility	Drug delivery methods	Relatively bioavailability (intramuscular injection)	Converted to dihydroartemisinin (in animals)	Blood-brain/blood-placenta barriers	Tissue distribution	*t*_1/2_	Metabolism	Disease
Artemisinin	Low solubility in water and in oil	Oral/intravenous/intramuscular/rectal/parenteral administration	32%	N/A	Blood-brain and blood-placenta barriers	N/A	1.9–2.6 h (oral)	Cytochrome P450 isozyme	Diabetes-insulin resistance-browning of white fat diabetes-islet ß cells-promoting insulin secretion/protect pancreatic ß cells/damage ß cells diabetic kidney disease diabetic retinopathy diabetic cardiovascular disease diabetic cognitive impairment
Artemether	Highly lipid-soluble	Intramuscular administration	54%	3.7–12.4%	Blood-brain barriers	(iv. 5 min) High: Brain Moderate: Heart, lung and skeletal Muscles Low: liver, kidney	74.6 min (stomach acid conditions) 1.1–3.1 (oral) 22.4–34.6 h (im.)	Cytochrome P450 isozyme	Diabetes-insulin resistance-browning of white fat/inhibiting inflammatory response diabetes-islet ß cells-promoting insulin secretion/protect pancreatic ß cells Diabetes-α-β Transdifferentiation (+) (−) diabetic kidney disease diabetic cognitive impairment
Dihydroartemisinin	Lipid-soluble	Intramuscular administration	85%	N/A	Blood-brain barriers	N/A	1.15–2.37 h (iv.) 30 min (im.) 37 min (oral)	Uridine glucuronyltransferase	Diabetes-insulin resistance-browning of white fat diabetes-islet ß cells-damage ß cells Diabetes-α-β Transdifferentiation (+) diabetic retinopathy
Artesunate	Highly water-soluble	Intravenous/intramuscular/rectal/oral administration	105%	25.3–72.7%	Blood-brain barriers	(iv. 5 min) brain, liver, kidney, testicle, muscle, fat, heart, serum, eyeball, spleen, lung (after 1 h) High: brain, fat, intestine, serum	43 min (oral) 1.5–11. 5 min (iv.) 30–41 min (im.)	Common esterases	Diabetes-insulin resistance-browning of white fat diabetes-islet ß cells-promoting insulin secretion/protect pancreatic ß cells Diabetes-α-β Transdifferentiation (+) (−) diabetic kidney disease diabetic retinopathy diabetic cardiovascular disease diabetic cognitive impairment
Arteether	Highly lipid-soluble	Intramuscular administration	34%	3.4–15.9%	NO	(im.24 h) High: Intestinal tracts, liver, kidneys and spleen Moderate: brown fat and brain Low: Heart, testes, muscle and residual carcass	200 min (stomach acid conditions) 23 h (im.)	Cytochrome P450 isozyme	Diabetes-α-β Transdifferentiation (+)

## Type 2 Diabetes

It is now generally accepted that IR and progressive damage to islet cell function are the fundamental pathological mechanisms of T2DM (Li et al., 2014). IR is often the first pathological manifestation of T2DM and accompanies the entire disease process (Kim et al., 2019; Wang et al., 2020). Before the diagnosis of T2DM, patients often have long-term IR, and the body compensates by secreting more insulin. As the disease progresses, islet ß-cell function is impaired, while glucose metabolism is severely decompensated, and eventually T2DM develops. Therefore, the crucial goals for treating T2DM are mainly to improve IR and islet cell function. In Fu’s research, islet function and IR were significantly improved in male db/db mice treated for 4 weeks with 100 mg/kg or 200 mg/kg artemether (Fu et al., 2020). Many studies have shown that artemisinin and its derivatives have great potential in the treatment of T2DM, whether in the early stage or late stage of the disease.

### Insulin Resistance

IR is a precursor of T2DM caused by decreased glucose metabolism and utilization efficiency of liver, adipose, skeletal muscle, and other tissues for various reasons (Czech, 2017; Yazıcı and Sezer, 2017; Petersen and Shulman, 2018). Many conditions can induce IR, with obesity being one of the most common and main precursor conditions. Obesity can induce disorders of adipose metabolism, followed by free fatty acid accumulation and chronic inflammation, consequently leading to IR and accelerating the progression of T2DM. Naturally, weight loss can effectively improve adipose metabolism, increase insulin sensitivity, and improve the basic condition of T2DM patients. According to clinical observations, weight loss-induced improvements in glycemia are most likely to occur early in the natural history of T2DM, when obesity-associated IR has caused reversible ß-cell dysfunction, but insulin secretory capacity remains relatively preserved (Schauer et al., 2016; Steven et al., 2016).

The effects of artemisinin and its derivatives on IR have attracted increasing attention. In 2010, Goto et al. found that eating terpenoids daily might be useful for the management of obesity-induced metabolic disorders, such as T2DM, hyperlipidemia, and IR (Goto et al., 2010). In subsequent studies, artemisinin-induced improvements in glucose tolerance test (GTT) and insulin tolerance test (ITT) results and a decrease in the IR index (HOME-IR) have been observed simultaneously (Kim et al., 2016; Lu et al., 2016; Li et al., 2017; Guo et al., 2018). These results confirm the important role of artemisinins in improving IR. Notably, many studies have reported that artemisinin and its derivatives can increase insulin sensitivity and improve IR. Artemisinin and its derivatives can also reduce food intake and the rate of body weight increases caused by a high-fat diet (HFD) (Lu et al., 2016; Guo et al., 2018; Fu et al., 2020). It seems that the antiobesity effect of artemisinins may be one of the most important ways they alleviate IR.

#### Acting on Adipocyte Production and Differentiation

Reducing adipogenesis and altering the direction of adipocyte differentiation can effectively alleviate obesity and the IR caused by obesity. Currently, strong anti-adipogenesis effects of artemisinins have been reported. In both 3T3-L1 adipocytes and human primary adipocytes, artemisinin inhibited the generation of intracellular lipids, reduced triglyceride (TG) levels, and lowered glyceraldehyde-3-phosphate dehydrogenase activity in a dose-dependent manner, indicating that artemisinin affects adipocyte differentiation but does not change the number of human adipocytes (Lee et al., 2012a; Jang, 2016). Glucose transporter-4 (GLUT4) is a marker gene of late-stage preadipocyte differentiation, and vascular endothelial growth factor (VEGF) is abundantly expressed in mature adipocytes but not in preadipocytes, and these markers are widely used to evaluate the development of adipocytes (Miyazawa-Hoshimoto et al., 2005). Investigators confirmed that the antiadipogenic effects of artemisinic acid alter human adipose tissue-derived mesenchymal stem cell differentiation by reducing GLUT4 and VEGF levels. In addition, the overinduction of hepatic sterol regulatory element-binding protein 1 (SREBP1) (Tu et al., 2012; Han et al., 2015) and carbohydrate-responsive element-binding protein (ChREBP) in both ob/ob mice and HFD mice indicated the overproduction of glucose (Benhamed et al., 2012; Abdul-Wahed et al., 2017). Interestingly, the administration of *A. annua* extract decreased the nuclear levels of SREBP1 and ChREBP and increased the phosphorylation of acetyl-CoA carboxylase (ACC), and it ameliorated hepatic steatosis and IR. These findings indicate the reversal of hepatic *de novo* lipogenesis and lipid accumulation (Kim et al., 2016).

CCAAT/enhancer-binding proteins (C/EBPs) and peroxisome proliferator-activated receptors (PPARs) are widely valued as dietary lipid sensors that control energy homeostasis (Dubois et al., 2017; Gross et al., 2017; Blüher, 2019). Knocking out or reducing the expression/activity of C/EBP-α or PPAR-γ by the pharmacological inhibitor of each or by siRNA transfection inhibits lipid accumulation during adipocyte differentiation (Linhart et al., 2001; Lehrke and Lazar, 2005; Rosen and MacDougald, 2006). Jang et al. demonstrated that artesunate at 5 μM precisely reduced the expression of C/EBP-α and PPAR-γ during adipocyte differentiation (Jang, 2016). Notably, another study attempted to elucidate the possible mechanism underlying the artemisinic acid-mediated effects by incubating artemisinic acid or artesunate with human adipose tissue-derived mesenchymal stem cells for 15 days. The results show that the development and differentiation of adipocytes were inhibited by the suppression of the master regulators C/EBP δ and PPARγ during adipogenesis (Lee et al., 2012a). Furthermore, decreased expression of the C/EBP δ gene was related to the inhibition of Jun N-terminal kinase (JNK) and activator protein 1 (AP-1) upon artemisinic administration when PPARγ was suppressed and the lower expression of genes that participate in controlling adipocyte fatty acid metabolism, including lipoprotein lipase (LPL), fatty acid translocase (CD36) and liver X receptor α (LXR α) (Liu et al., 2007; Lee et al., 2012a). Artemisinic acid also inhibits the expression and activity of gelatinase matrix metalloproteinase (MMP)-2, which is important to the development of adipose tissue (Croissandeau et al., 2002; Lee et al., 2012a). These results show that artemisinins inhibit adipocyte formation and differentiation by suppressing the master regulators C/EBPs and PPAR γ and related molecules to improve IR.

IR is also attributed to differentiated adipocytes, which synthesize and release an array of adipokines, including fatty acid synthase (FAS), leptin, perilipin A, and the phosphorylation levels of signal transducer and activator of transcription-3 (STAT-3) (Morrison and Farmer, 2000; Fève, 2005; Jang, 2016). Increasing evidence suggests that adiponectin and leptin are involved in the endocrine control of energy homeostasis, and the leptin/adiponectin ratio is often used as a surrogate marker for insulin sensitivity (Drevon, 2005; Trujillo and Scherer, 2005; Finucane et al., 2009; Yadav et al., 2013). RT-PCR analysis further revealed that artesunate reduced the insulin- and FBS-induced mRNA expression of FAS and leptin and enhanced the mRNA levels of adiponectin (Jang, 2016). These findings may suggest that artemisinins lower adipokine levels and attenuate IR by affecting the leptin/adiponectin ratio.

#### Activation of Brown Fat and Browning of White Fat

Adipose tissue is metabolically active and can be classified as white, brown and beige adipose tissue based on the morphology, physiology, and function (Boss and Farmer, 2012; Xu et al., 2018). The imbalance of brown fat and white fat leads to abnormal adipose accumulation, which in turn produces a series of metabolic diseases. The activation of brown fat and browning of white fat are the two main sources of adaptive heat generation and important output of energy expenditure (Cypess and Kahn, 2010; Boss and Farmer, 2012). Interestingly, some studies reported that artemisinin and its derivatives can activate brown adipose tissue and brown white adipose tissue, effectively inhibiting abnormal adipose accumulation and ameliorating IR. Lu et al. have identified artemether as an activator of browning and thermogenesis *in vitro*, which significantly enhances the metabolism of mice, as indicated by the insulin tolerance test (ITT) and glucose tolerance test (GTT) results (Lu et al., 2016). To further evaluate the pharmacological potential, Lu et al. found that artemether and other artemisinin derivatives induce C3H10T1/2 cell browning by activating the p38 mitogen-activated protein kinase (MAPK)/activating transcription factor-2 (ATF2) axis and deactivating the Akt/mTOR pathway, which has suggested to be involved in various anabolic and catabolic processes (Cai et al., 2016; Lu et al., 2016; Fischer et al., 2020; Toda et al., 2020). Moreover, one-step qPCR suggested that the relative mRNA levels of browning-related genes, such as PR domain containing 16 (PRDM16), uncoupling protein 1 (UCP1) and peroxisome proliferator-activated receptor γ coactivator-1α (PGC-1α), were elevated after treatment with artemether, indicating that the increased thermogenesis in brown fat may also cause weight loss and enhance the metabolism in artemether-treated mice (Lu et al., 2016).

#### Inhibiting the Inflammatory Response

IR is closely associated with chronic inflammation (Brenachot et al., 2017; Caprio et al., 2017; Reilly and Saltiel, 2017; Saltiel and Olefsky, 2017; Wu and Ballantyne, 2020). Abnormal accumulation of adipose and the increased release of free fatty acids, which can be internalized by hepatocytes, consequently leading to chronic inflammation (Kamari et al., 2011; Saltiel and Olefsky, 2017; Shimobayashi et al., 2018). Moreover, preadipocytes and macrophages release proinflammatory cytokines, including tumor necrosis factor α (TNF-α) (Hotamisligil et al., 1993), interleukin 6 (IL-6) (Rotter et al., 2003; Sopasakis et al., 2004), interleukin 1β (IL-1β) (Tateya et al., 2013), and monocyte chemoattractant protein 1 (MCP-1), and induce IR in target cells (Wellen and Hotamisligil, 2003; Bastard et al., 2006; Olefsky and Glass, 2010). Due to the interference of artemisinic acid with adipogenesis, artemisinic acid significantly attenuated the increased secretion of TNF-α and IL-6 in undifferentiated human adipose tissue-derived mesenchymal stem cells, influencing the inflammatory state (Lee et al., 2012a). In addition, cyclooxygenase-2 (COX-2) is an inducible enzyme that is expressed at low levels in normal tissues but is highly expressed when cells are stimulated by inflammation (Ferrer et al., 2019). Kim’s team found that *A. annua* leaf extract significantly improved ITT and GTT results and decreased COX-2 levels in HFD-fed mice (Kim et al., 2016). Recently, investigators also found that artemether can promote AMP-activated protein kinase (AMPK) activity and downregulate the expression of inflammatory factors to reverse the pathological state in db/db mice (Fu et al., 2020). NF-κB plays a key role. Many studies have shown that inhibiting the abnormal activation of the NF-κB signaling pathway by various stimuli, such as endogenous advanced glycation end products (AGEs), can reduce the inflammatory response, which has been proven to contribute to anti-IR effects (Shoelson et al., 2006). In 2001, researchers understood that the anti-inflammatory drug salicylic salicylate greatly ameliorates IR and the symptoms of diabetes via the IKKβ/NF-κB axis (Yuan et al., 2001). Surprisingly, many studies have shown that artemisinin and its derivatives exhibit a strong anti-inflammatory effect by targeting the NF-κB signaling pathway, which is a molecular mechanism critical for the state of chronic inflammation in IR and T2DM; however, direct evidence showing that artemisinins reverse IR through NF-κB is lacking (Baumgart and Sandborn, 2007; Xu et al., 2007; Duckworth et al., 2009; Chen et al., 2010; Lee et al., 2012b; Yazıcı and Sezer, 2017). In summary, through a literature review, we found that artemisinins have anti-inflammatory effects and relieve IR. Based on the common pathological state of chronic inflammation in diabetes and IR, it is speculated that the anti-inflammatory effects of artemisinins have a causal relationship with attenuated IR (Fu et al., 2020).

### Restoring Islet ß-Cell Function

Obesity-related IR causes reversible ß-cell dysfunction, which in turn affects the secretion of insulin (Steven et al., 2016). However, long-term IR or hyperglycemia can cause irreversible damage to islet cells and lead to a complete failure of insulin secretion, which is an important feature of T2DM progression. Most agree that protecting ß cells is beneficial to insulin secretion and glucose control and slows the T2DM process (Marrano et al., 2020). Increasing evidence indicates that artemisinin and its derivatives have the potential to alleviate T2DM by restoring islet ß-cell function.

On the one hand, the effect of artemisinins on promoting insulin secretion is gradually recognized (Kim et al., 2016; Li et al., 2017; Guo et al., 2018; Xiang et al., 2019). In 2012, after treatment with ethanolic leaf extracts (100 and 200 mg/kg) of *A. annua*, Wistar rats showed a significantly reduced glucose concentration, and there were no adverse effects on liver function, hematological indices, or testosterone levels (Eteng et al., 2013). Kim and the team proved that *A. annua* inhibited α-glucosidase activity in a dose-dependent manner even more effectively than acarbose, a known antidiabetic drug, in HFD mouse models (Kim et al., 2016). It has also been reported that artemisinin and its derivatives attenuate diabetic hyperglycemia by increasing insulin secretion, which has been observed not only in rats and mice but also in human islets (Li et al., 2017). Moreover, the unbalanced ratio of insulin, glucagon, and somatostatin content was also reversed by the administration of artemether in islets (Guo et al., 2018). The increase in insulin concentration was accompanied by a decrease in proglucagon, glucagon, and processed glucagon peptides in αTC1 cells treated with artemether and its analogs, namely, dihydroartemisinin, arteether, and artesunate, for 72 h, except for the deoxyarteether-treated group, in which the insulin expression did not change (Li et al., 2017). These findings demonstrated that, although their physical and chemical properties are very similar, artemisinin and its derivatives show differences in pharmacological effects and/or effectiveness. The applications of different kinds of artemisinins require further study. After in rats injected with streptozotocin (STZ) received 4 weeks of 300 mg/kg/d artemisinin treatment, upregulated insulin levels and the insulin-like growth factor binding protein one gene (Igfbp1) were observed (Xiang et al., 2019). Furthermore, artemisinin has long-term effects on diabetes treatment, which is reflected by the improved hemoglobin A1c (HbA1c) levels (Han et al., 2019). As for the specific mechanism of artemether, through pull-down assays, artemether was found to interact with the protein gephyrin, and it strengthens gephyrin expression, increasing the expression of P2rx3, Vamp1, and Nrxn3 genes, resulting in the activation of the GABA_A_ receptor complex and GABA signaling, subsequently increasing insulin secretion and inhibiting glucagon secretion (Soltani et al., 2011; Purwana et al., 2014; Li et al., 2017). When gephyrin was knocked down, single-cell analysis of the image data revealed a high correlation between increases in gephyrin and insulin, which was basically abolished, in artemether-treated cells (Li et al., 2017). Yu et al. reported that artesunate reversed the suppressed state of insulin secretion caused by IL-1β in rat islets after stimulation with 16.7 mmol/L glucose, while artesunate alone did not affect insulin secretion in normal rat islets (Yu et al., 2016). This finding indicates that artesunate may play the role of “balancer” and thus helping to achieve homeostasis. Moreover, in ß-cells, artesunate upregulated the expression of SIRT1, which plays a key role in glucose/lipid metabolism, and deacetylated lysine residues on various transcription factors, such as FOXO and PGC-1α, ultimately stimulating insulin secretion (Monteiro and Cano, 2011; Lai et al., 2012; Yu et al., 2016; Han et al., 2019).

On the other hand, studies have shown that artemisinin and its derivatives play roles in protecting islet ß cells. Db/db mice were treated with 200 mg/kg artemether for 2 weeks, and Guo’s team observed that artemether significantly reversed pancreatic ß-cell damage, which was reflected in improved islet morphologies, ameliorated islet vacuolar degeneration, a reduced apoptosis rate of pancreatic ß cells and increased islet cell numbers and size (Guo et al., 2018). Hence, artemether has generated intense interest for use in strategies designed to regenerate functional ß cells toward a cure for diabetes (Thorel et al., 2010; Chera et al., 2014). Upon further research, it was reported that artemisinins triggers ß-cell-like induction of neogenesis following streptozotocin (STZ)-induced ß-cell death by indirectly activating the GABA signaling pathway in cell, zebrafish larva, and wild-type mouse models (Ben-Othman et al., 2017; Li et al., 2017). Moreover, artesunate can stimulate SIRT1 expression, which not only improves insulin levels but also protects pancreatic ß cells (Kitada and Koya, 2013). An abundance of evidence confirms that artesunate can block the NF-κB pathway, inhibit inducible nitric oxide synthase (iNOS) expression and decrease nitric oxide (NO) production, conferring a protective effect on *ß* cells exposed to IL-1β (Bordone et al., 2006; Lee et al., 2009; Yang et al., 2012; Yu et al., 2016). The inhibition of the NF-κB signaling pathway may be an important target for artemisinin to protect islet cells. Artesunate can inhibit NF-κB nuclear translocation and reduce its transcriptional activity by promoting the deacetylation of p65, reducing the activity of IKK, and preventing IkB phosphorylation (Huxford et al., 1998; Dejardin, 2006; Xu et al., 2007; Yu et al., 2016). In addition, the protective effects of artemisinin on islet ß cells is also reflected by the reversed suppression of cell proliferation at the same time that inhibitor of DNA binding 1 (ID1) and cyclin-dependent kinase inhibitor 1A (CDKN1A) levels are increased by STZ (Xiang et al., 2019).

However, some subsequent papers failed to replicate these findings. van der Meulen et al. found that artemether-treated islets showed an obvious pattern of speckles or fragmentation in the red channel after 72 h, which indicated that ß-cell health had declined in male islets from Ins1-H2B-mCherry × Gcg-Cre × Rosa26-stop-YFP triple transgenic mice (van der Meulen et al., 2018). In contrast to the roles they play upon inflammatory factor-induced ß-cell damage, artemisinins may participate in the ß-cell damage specifically induced by palmitate. In contrast to Yu’s conclusion, Chen et al. found that artemisinin and dihydroartemisinin cause the deterioration following pancreatic ß-cell damage in palmitate-induced INS-1 and MIN6 cells by triggering ER stress. The expression levels of ER stress-related mRNA (GRP78, CHOP, PDI) in the artemisinin and dihydroartemisinin groups were both increased in a dose-dependent manner (Chen et al., 2020). The researchers of this study concluded that relatively high concentrations of artemisinin and dihydroartemisinin may cause damage to pancreatic cells in obese patients but not in healthy individuals.

### A Controversial Mechanism: α-Cell to ß-Cell Transdifferentiation

Although T1DM and T2DM are fundamentally different diseases, both are associated with a deficiency in functional ß cells. It has been suggested that the defect in the quality of ß cells in diabetes is not due to the death of ß cells but to the dedifferentiation or transdifferentiation of ß cells (Talchai et al., 2012; Spijker et al., 2013). In addition, phenotypic changes of ß cells promote transdifferentiation to other pancreatic endocrine cells (mainly α cells and δ cells) (Spijker et al., 2013; Cinti et al., 2016). The possibility of curing T2DM by reversing the phenotypic changes that cause ß-cell identity loss or by promoting other cells to transdifferentiate into ß cells is gradually being recognized and considered. Specifically, α cells are attractive starting points for transdifferentiation protocols, as they are developmentally closely related to ß cells (Thorel et al., 2010; Unger and Orci, 2010; Ye et al., 2015). Over the past decade, multiple studies have shown that pancreatic α cells can transdifferentiate into ß cells or ß-like cells after deletion of the α cell-specific transcription factor Arx (Courtney et al., 2013; Wilcox et al., 2013; Chakravarthy et al., 2017), overexpression of transcription factors necessary for ß-cell differentiation, such as Pax4 (Collombat et al., 2009) or Pdx1 and Mafa (Matsuoka et al., 2017), or after extreme ß-cell loss (Thorel et al., 2010). Li et al. found that artemisinins were able to convert glucagon-producing α cells into insulin-producing ß cells (Li et al., 2017). The expression of Arx was significantly downregulated, while Pax4 and Mnx1 were elevated in the artemisinins (artemether, dihydroartemisinin, arteether, and artesunate) groups. They found that antimalarial drugs from the artemisinin family (particularly artemether) induced the conversion of α ells into ß-like cells by enhancing GABA signaling not only in mouse cell lines but also in mice (using lineage tracking), rats, and zebrafish *in vivo*. Vieira et al. suggested that artemether acts through its interaction with gephyrin, which potentiates GABA signaling and induces Arx translocation from the nucleus to the cytoplasm, thereby leading to its inactivation and the consequent conversion of α cells into ß-like cells (Vieira et al., 2017). Reducing the abundance of both proglucagon and processed glucagon peptides inhibited glucagon secretion under low-glucose conditions, thereby triggering the loss of α-cell identity. After artemether treatment with key ß cell-specific genes, such as GNAS and ABCC8, were profoundly upregulated, whereas α cell-specific genes, including EIF4A1, CRYBA2, PDK4, and MUC13, were significantly downregulated in human islets (Li et al., 2017).

However, some researchers have questioned this phenomenon. Although alterations in the identity of α cells and/or ß cells have been observed, and there no direct evidence of α cell to ß cell transdifferentiation has been observed in subsequent experiments. Li’s study explored whether the identity and functional maturation of ß cells are influenced by 1 mg/ml artesunate in female NOD mice. ß-cell mass was significantly increased, whereas α-cell mass was not altered after artesunate treatment when Ins1, Ins2, MafA, Ucn3, and NeuroD1, which are essential for maintaining the identity and functional maturation of ß cells, were dramatically increased (Nishimura et al., 2015; Li et al., 2019d). Interestingly, the expression of the endocrine progenitor marker Ngn3 was decreased in artesunate-treated islets (Li et al., 2019d). Ackermann et al. used Glucagon-CreERT2; Rosa-LSL-eYFP male mice as models, in which >90% of α-cells were labeled, which enabled the accurate quantification of mature α cell to ß cell transdifferentiation. The investigators treated the mice for 3 months with 1 mg/ml artesunate. At the end of this period, the results revealed no changes in the fractions of insulin+/YFP + cells, ß-cell area, islet number, or proportion of pancreatic area composed of islets between the treatment and control groups. Hence, there were no indications of a naturally occurring, slow transdifferentiation process *in vivo* (Ackermann et al., 2018). van der Meulen et al. reported that a 3 days artemether treatment of islets from Ins1-H2B-mCherry × Gcg-Cre × Rosa26-stop-YFP triple transgenic reporter male mice caused the sustained loss of identity across all islet endocrine cell types. The expression of other α-cell genes, including Gcg, Mafb, and Irx1, was also downregulated, suggesting a general loss of α-cell identity, and some mature ß-cell markers, including Ucn3, Mafa, Pdx1, and Slc2a2, and two δ-cell markers, somatostatin (Sst) and Hhex, were significantly inhibited. Indeed, the expression of Ins1 and Ins2 was downregulated by >10- and >100-fold, respectively. These results confirmed that artemether does not selectively inhibit Arx but causes broad inhibition of α cell, ß cell, and δ cell-specific transcription factors (van der Meulen et al., 2018). Brenda Marquina-Sanchez et al. pointed out that any sample carryover or cross-contamination that occurs in droplet-based single-cell RNA-seq greatly affects the results of the experiment, and therefore, the results need to be effectively corrected. They developed a method that combined standardized reference cells as spike-in controls with a computational decontamination algorithm to eliminate differences and obtain more accurate conclusions. With this method, in addition to increased insulin expression, the downregulation of α cell-specific genes and upregulation of key ß-cell genes were observed in the α cells of mouse and human islets. In subsequent experiments, they also found that the effects of artemether on ß cells in pancreatic islets were species-specific, causing more species-dependent gene expression changes (Marquina-Sanchez et al., 2020). In contrast to the decrease in ß cells in the mouse model, human islet ß cells showed a slight increase. Similarly, the expression of key ß-cell genes, such as INS1/2, SLC2A2, ISL1, GCGR, UCN3, and SCG5, decreased in the mouse models but increased in the human islets. In addition, SPP1 and Igf1r have also undergone inconsistent changes. These data suggest that drug effects are species-dependent.

In summary, it can be seen from these results that most of the experiments did not directly lead to the conclusion that artemisinin can achieve α-cell to β-cell transdifferentiation, but many experiments revealed the emergence of functional β cells or/and the restoration of β-cell identity. Unfortunately, some experimental results showed that artemisinin does not lead to transdifferentiation and may further damage β cells. Combined with the results of studies on the characteristics of artemisinin and its derivatives and comparing the relevant experimental process and results, we speculate that sex, food intake, and objective pollutants may be the reasons for the differences in the experimental results. Although there is no direct evidence that sex plays a role in artemisinin-promoting transdifferentiation, females have higher bioavailability than males after administration of artemisinins. Since most experiments use single-sex animal models or cells, it would be interesting and is necessary to study and compare the effects of sex (Navaratnam et al., 2000). On the one hand, food intake can affect the absorption and metabolism of artemisinin (Ashton et al., 1999); on the other hand, it can affect the intestinal flora, and artemisinins may affect the intestinal flora and thereby modulate the state of the overall internal environment. Brenda et al suggested that species differences and the presence of pollutants in general methods are also noteworthy (Marquina-Sanchez et al., 2020), which shows that we may be able to obtain more precise results and obtain more accurate conclusions by improving experimental technology.

## Type 2 Diabetes Mellitus-Related Complications

### Diabetic Kidney Disease

Diabetic kidney disease (DKD) is a serious complication of diabetes (Sharaf El Din et al., 2017; Tanabe et al., 2017; Tesch, 2017; Uwaezuoke, 2017; Wu et al., 2018b). The early clinical manifestations of DKD are decreased glomerular filtration, followed by increased arterial blood pressure, proteinuria, and fluid retention, which ultimately lead to renal failure (Egido et al., 2017; Tanabe et al., 2017; Wu et al., 2018b). Glomerular hypertrophy, thickening of the glomerular and tubular basement membrane, and accumulation of extracellular matrix in the mesangial area can be observed in the early stages of DKD. The late pathological features are glomerular and tubular interstitial fibrosis (Dai et al., 2017; Kitada et al., 2017). Recently, it was reported that artemisinin and its derivatives may be a promising therapy for DKD.

The symptoms, signs, and pathological changes of diabetic nephropathy were attenuated in artemisinin-treated rats. Urinary albumin excretion was significantly decreased, and serum total protein (TP) and albumin (ALB) levels were restored in the artemisinin group compared to the levels in the diabetic without treatment group (Han et al., 2019; Wang et al., 2019b; Xiang et al., 2019). In addition, tubular injury biomarkers also show a downward trend, and the decreased N-acetyl-β-D-glucosaminidase (NAG), neutrophil gelatinase-associated lipocalin (NGAL) and kidney injury molecule-1 (Kim-1) levels explained the effect of artemether in proteinuria reduction (Wang et al., 2019b). Notably, blood urea nitrogen (BUN), creatinine (Cr), creatinine clearance rate (Ccr) and urinary albumin excretion rate (UAER) were downregulated in the artemisinin and its derivatives treatment group, and glomerular hypertrophy and hyperplasia, glomerular basement membrane (GBM) and tubular basement membrane (TBM) thickening, glomerular capillary dilatation, foot process width (FPW) broadening and extracellular matrix accumulation, which are characteristics of mice with DKD, were all attenuated (Zhang et al., 2014a; Zhang et al., 2014b; Zhang et al., 2014c; Zhou et al., 2014a; Zhou et al., 2014b; Zhou et al., 2014c; Nie et al., 2015; Xiang et al., 2019; Zhang et al., 2020). In addition, diabetic symptoms such as polydipsia, polyuria, urinary glucose, increased food intake and weight loss, were all reversed by artemisinins (Han et al., 2019; Wang et al., 2019b; Zhang et al., 2020).

Recent studies have demonstrated that metabolic alterations and mitochondrial dysfunction play critical roles in DKD initiation and progression (Forbes and Thorburn, 2018). The diabetic kidney is characterized by incomplete glucose oxidation and enhanced fatty acid utilization. Artemether treatment increased the respiratory exchange ratio (RER) but did not affect total energy expenditure, indicating that artemether shifted the energy metabolic substrate from lipids and proteins to glucose (Choi et al., 2015). To further address the role of artemether on mitochondrial function, treating male db/db mice with 0.67 g/kg artemether for 12 weeks, Han et al. reported first that artemether inhibited the production of renal mitochondrial hydrogen peroxide (H_2_O_2_), reduced serum and urine H_2_O_2_ levels, and regulated the expression of renal cortex- and mitochondrial-related proteins. Specifically, PGC-1α, which can stimulate MPC1 transcription, MPC1, and MPC2, which are both carriers that facilitate pyruvate transport into mitochondria, are all enhanced with artemether therapy (Choi et al., 2014; Rabinovitch et al., 2017; Han et al., 2019). Similar results were replicated in another experiment. Apart from restoring PGC-1α, MPC1, and MPC2 levels, the expression of pyruvate dehydrogenase kinase 1 (PDK1) also decreased in the artemether group compared to the levels in the diabetic STZ mice, indicating enhanced pyruvate oxidation in mitochondria (Wang et al., 2019b). Oxidative stress is a crucial pathogenesis of DKD. Artemisinin attenuates renal damage in DKD rats by suppressing transforming growth factor-β1 (TGF-β1) regulation, increasing antioxidant proteins heme oxygenase-1 (HO-1) and NADPH quinone acceptor oxidoreductase 1 (NQO1) and activating the nuclear factor-erythroid 2-related factor 2 (Nrf2) signaling pathway (Jha et al., 2016; Akash et al., 2018; Zhang et al., 2020).

Amelioration of DKD by inhibiting the inflammatory response and preventing the inflammatory pathway is gradually being recognized and appreciated. A high-glucose environment and oxidative stress state induce the transfer of protein kinase C (PKC) from the cytoplasm to the cell membrane in nephrocytes; that is, the translocation of PKC is induced, resulting in the phosphorylation of the transcription factors AP-1 and NF-κB, which can upregulate the expression of downstream genes, thereby increasing the production of a large number of cytokines and growth factors, causing renal cell hypertrophy and proliferation, glomerular basement membrane thickening and cell-matrix accumulation. Zhang et al. proved that intraperitoneal injection of artemisinin can inhibit the activation of PKC, downregulate AP-1 synthesis-related genes c-jun and c-fos expression, and further decrease the expression of AP-1 and NF-κB, ultimately alleviating renal pathological changes induced by high glucose and reactive oxide (Zhang et al., 2014b; Zhou et al., 2014a; Zhou et al., 2014b; Zhou et al., 2014c). Nie et al. speculated that the efficacy of 30 mg/(kgd) artesunate might be similar to that of 10 mg/(kgd) enalapril in protecting renal function by lowering the expression of TLR4 and IL-8 (Nie et al., 2015). Sun et al. reported that artesunate conferred protective effects on HG-induced HBZY-1 cells through the toll-like receptor 4 (TLR4)/NF-κB/nod-like receptor protein 3 (NLRP3) inflammasome pathway (Sun et al., 2018). The results suggested that artemisinins might be potential therapeutic agents for DKD treatment.

During the onset and progression of diabetic nephropathy, the renin-angiotensin system (RAS) in kidney tissue plays an important role. In the hyperglycemic state, RAS in renal tissue can be activated, causing an increase in angiotensin II (AngII) levels in renal tissue, leading to the accumulation of platelet-derived growth factor (PDGF) and an imbalance of matrix metallopeptidases (MMPs)/tissue inhibitor of metalloproteinases (TIMPs). In the artemisinin-treated group, decreased MMP-2 protein levels and increased expression of PDGF-B and TIMP-2 in the glomeruli were obviously reversed. In addition, artemisinin significantly downregulated the expression of fibronectin (FN) and collagen IV significantly reducing the accumulation of the extracellular matrix in glomeruli and enhancing renal function (Zhang et al., 2014a; Zhang et al., 2014c).

Next-generation sequencing for ditag genome scanning (DGS) was applied to study the effect of artemisinins on DKD (Kelly et al., 2013; Rudnicki et al., 2015). Using a microarray, Brennan et al. found that Tgfbi and Ark5 were induced by TGF-β1 and were also upregulated in human DKD (Brennan et al., 2012). Using RNA sequencing, Xiang et al. examined the profile of differentially expressed genes following the administration of artemisinin. They found that 69 gene expression levels were different between the normal samples, STZ samples, and artemisinin treatment samples. Specifically, 38 genes, including insulin-like growth factor binding protein 1 (Igfbp1), sulfotransferase 1A1 (Sult1a1) and six-transmembrane epithelial antigen of prostate 4 (Steap4), were increased after artemisinin treatment, and 31 genes, including 3-hydroxy-3-methylglutaryl-CoA synthase 2 (Hmgcs2), ID1, and CDKN1A showed a downtrend in the artemisinin group compared to the STZ group. These identified genes were also related to a list of Gene Ontology (GO) terms and the Kyoto Encyclopedia of Genes and Genomes (KEGG) pathways. For example, the pathways involve “complement and coagulation cascades,” which have been reported to play important roles in the progression of DKD (Wang et al., 2016c), and the “p53 signaling pathway” and “TGF-β signaling pathway,” which are closely associated with DKD (Zhang et al., 2011; Wang et al., 2016a; Mukhi et al., 2017; Xu et al., 2017a; Shelbaya et al., 2018; Sheng et al., 2018; Xiang et al., 2019). These results indicate promising targets in the treatment of DKD with artemisinins.

### Cognitive Impairment Related to Type 2 Diabetes Mellitus

There is increasing evidence that diabetes predisposes individuals to cognitive decline, even leading to dementia (Biessels et al., 2006; Grünblatt et al., 2011; Wong et al., 2014). It has been postulated that Alzheimer’s disease (AD) may represent the consequence of a distinct form of brain-specific IR and impaired glucose regulation (Xu et al., 2010; Akter et al., 2011; de la Monte, 2012; Shuba and Karan, 2012). However, the effectiveness of the current treatment for T2DM-related cognitive dysfunction is far from sufficient. Moreover, the insulin treatment itself can exacerbate cognitive impairment (Ott et al., 1999). In the progression of T2DM, the inflammatory response and oxidative stress caused by high glucose are the bridges between diabetes and cognitive impairment (Dinel et al., 2011). Recently, the great potential of artemisinin and its derivatives in reversing diabetic cognitive impairment has gradually received attention.

HFD/STZ administration induces a state of neuroinflammation, as indicated by the infiltration of inflammatory cells and elevated protein and mRNA expression levels of TNF-α, IL-6, and iNOS, which can be reversed by artemisia extract (Gaspar et al., 2016; Albasher et al., 2020). Through a literature review, we found more possible but unproven mechanisms. The activation of the NF-κB signaling pathway is closely related to the release of proinflammatory factors, while inhibiting inhibitor of kappa B (IκB) α can enhance the learning ability and memory of diabetic rats (Datusalia and Sharma, 2016). In Shi’s study, artemisinin decreased the neuritic plaque burden and improved AD symptoms by inhibiting NF-κB activity and NALP3 inflammasome activation in APPswe/PS1dE9 double transgenic mice but not diabetic rats (Shi et al., 2013). *In vitro* experiments were performed with lipopolysaccharide (LPS), which is an essential component of the Gram-negative bacterial cell wall and is most commonly used to activate astrocytes (Chen et al., 2015; Zhang et al., 2016). Artemisinins (artemisinin, artesunate, and artemether) showed an anti-inflammatory action in an LPS-induced BV-2 mouse microglial cell line by interfering with IκB/NF-κB signaling, releasing fewer pro-inflammatory mediators, such as NO, iNOS, prostaglandin E2 (PGE2), COX-2, microsomal prostaglandin E synthase-1 (mPGES-1), TNFα, IL-6, IL-1β, and the NLRP3 inflammasome complex, making them good candidates for decreasing the neuritic plaque burden and alleviating neurological inflammation disorders (Zhu et al., 2012; Shi et al., 2013; Badshah et al., 2015; Wang et al., 2015; Badshah et al., 2016; Okorji et al., 2016; Zuo et al., 2016; Irrera et al., 2017; Gugliandolo et al., 2018). In addition, suppressing the NF-κB pathway and inhibiting p38 phosphorylation and downstream kinase MAP kinase-activated protein kinase 2 (MAPKAPK2) are crucial ways for the anti-neuroinflammatory activity of artesunate (Okorji and Olajide, 2014). Another study discovered that dihydroartemisinin alleviates learning and memory and depression-like behavior and prevents neuronal degeneration in the hippocampal CA1, CA2, CA3, and DG regions by inhibiting the production of LPS-induced inflammatory mediators via the PI3K/AKT signaling pathway (Gao et al., 2020). These experiments point us in the direction of progress, but need to be validated in diabetic mice.

The interaction between inflammation and oxidative stress has been confirmed in T2DM (Teodoro et al., 2018). Hyperglycemia promotes oxidative stress, which is thought to play an important role in the progression of diabetes-related complications (Abdelfattah et al., 2020). According to the sensitivity of the brain to peroxide reagents, antioxidants have a place in diabetes-related cognitive impairment (Asmat et al. 2016). *A. annua* extract, which is an antioxidant similar to the antidiabetic standard drug metformin, upregulated cortical antioxidant enzymes (glutathione peroxidase, glutathione peroxidase 1, glutathione reductase, superoxide dismutase, superoxide dismutase 2, catalase) and downregulated lipoperoxidation levels [malondialdehyde (MDA), NO] in STZ diabetic rat models (Albasher et al., 2020). In other cognitive impairment diseases, artesunate enhanced the spatial learning ability of thioacetamide-induced male Wistar rats and shortened the escape latency and working errors (Wu et al., 2016). In addition to animal experiments, astrocytes and microglial cells have been studied because they play crucial roles in T2DM-mediated chronic inflammation and oxidative stress, maintaining brain homeostasis (Shi et al., 2018). Artemisinins significantly suppressed ROS release and played a protective role in Aβ25-35-induced PC12 cells and ammonia-treated SH-SY5Y cells (Okorji and Olajide, 2014; Badshah et al., 2015; Badshah et al., 2016; Wu et al., 2016). Furthermore, the protective effects of artemisinin on H_2_O_2_-induced SH-SY5Y and hippocampal neurons can also be achieved by activating AMPK signaling (Zhao et al., 2019). Another study confirmed that the antioxidant protection mechanism of artemether was dependent on Nrf2 in BV2 microglia and showed a downtrend in the production of ROS and elevated levels of the antioxidant protein NQO1 (Okorji et al., 2016). Artesunate showed a protective effect on ammonia-treated C6 astroglial cells by reversing oxidative stress, which subsequently led to elevated glutamate uptake/release activity, glutamine synthetase activity, intracellular glutathione (GSH) content, and Na^+^K^+^-ATPase activity (Wu et al., 2016). Treatment of cerebrovascular diseases with artesunate suppressed ROS production in CIRI mice by restoring Nrf2 protein expression and downregulating ROS-dependent p38 MAPK in these mice. In addition, the misfolding and aggregation of human islet amyloid polypeptide (hIAPP) and amyloid-β (Aβ) protein are closely related to T2DM and AD. In Xu’s study, while four compounds of artemisinins (artemisinin, dihydroartemisinin, artesunate, and artemether) regulated the glucose homeostasis of diabetes, they also had great inhibition and disaggregation effects against hIAPP (Xu et al., 2019). There is abundant evidence showing that artemisinins decrease the level of amyloid-β protein (Aβ), which accumulates in AD and attenuate neurocognitive deficits by inhibiting c-Jun N-terminal kinase (JNK) and activating the extracellular signal-regulated kinase (ERK) 1/2 pathway (Chong and Zheng, 2016; Xu et al., 2017b; Zeng et al., 2017). These findings provide new perspectives on the use artemisinins as inhibitors against amyloidosis-related diseases. Although we currently lack enough direct evidence to prove the specific effects of artemisinins on diabetic cognitive diseases, through a systematic assessment of the relationship between diabetes and cognitive impairment and the effects of artemisinin on other cognitive disorders, we speculate that artemisinin and its derivatives may prevent and treat diabetes-related cognitive impairment by relieving oxidative stress and suppressing inflammation status and propose some potential action points of the oxidative stress pathway and inflammatory pathway that deserve further exploration.

At high concentrations of glucose, *A. annua* extract significantly attenuated the vacuolation degeneration of neurocytes and prevented cortical apoptosis by enhancing the expression of anti-apoptotic marker Bcl2 and inhibiting the expression of proapoptotic marker Bax. In addition, the increased brain-derived neurotrophic factor norepinephrine and dopamine levels indicated neuroprotective efficiency in diabetes (Albasher et al., 2020). Interestingly, the protective results were replicated in an artesunate-treated traumatic brain injury (TBI) group, suggesting that artemisinins of broad spectrum protection of nerve cells (Gugliandolo et al., 2018).

Evidence indicates that the gut microbiome is a potential new target for Chinese herbal medicines in treating diabetes mellitus (Wang et al., 2018; Wu et al., 2019; Zhang et al., 2019). Currently, Liu et al. investigated the effects of dihydroartemisinin on the intestinal microbiome in mice. Dihydroartemisinin downregulates the “neurodegenerative diseases” and “infectious diseases” signaling pathways while upregulating “energy metabolism” and “nucleotide metabolism” as indicated by a KEGG signaling pathway enrichment analysis (Liu et al., 2020). However, the role of artemisinins in the intestinal flora of T2DM is still unclear and needs to be further explored.

### Diabetic Retinopathy (DR)

Diabetic retinopathy (DR) is a common complication of advanced T2DM and is largely related to two late-stage conditions: proliferative diabetic retinopathy and diabetic macular edema (Wong et al., 2016; Tan et al., 2017). Many factors regulate the proliferation and migration of endothelial cells through a series of molecular mediators (Amadio et al., 2016). Ge et al. directly proved that artesunate can inhibit retinal neovascularization and leakage by decreasing the increase in intercellular adhesion molecule-1 (ICAM-1) and MMP-9 protein in human umbilical vein endothelial cells (HUVECs) in a concentration-dependent manner under high-glucose conditions (Ge et al., 2019). In addition, VEGF can also destroy the blood-retinal barrier and lead to an increase in vascular permeability such that exuded fluid accumulates in the macula, which worsens retinal ischemia and hypoxia, seriously threatening vision (Crawford et al., 2009; Wong et al., 2016; Tan et al., 2017; Jiang et al., 2018). The current DR treatment strategies aim to control microvascular complications; therefore, the administration of intravitreal anti-VEGF drugs is currently the main means of therapy for early and advanced stages of DR (Cheung, Wong, and Wong, 2014; Wang and Lo, 2018). Interestingly, clinical and experimental studies demonstrated that artemisinins can reverse pathological angiogenesis and pathological exudation, which accelerates disease progression (Chen et al., 2003). Li et al. injected 80 μg of artesunate into five patients with retinal neovascularization, and upon following up at 52 weeks, found that retinal neovascularization, papilledema, and high intraocular pressure (IOP) were significantly relieved (Li et al., 2019a). A single intravitreal dose of 20 μg of artesunate was injected in rabbit and monkey models and 6 months later the artesunate had reversed retinal and iris neovascularization, vascular dilatation and tortuosity, macular edema, and fluorescein leakage. Current clinical trials have suggested that anti-VEGF therapy may represent a first-line therapy for proliferative DR treatment. In the present study, artesunate targeted VEGF and had greater anterior chamber penetrability and more durable efficacy than Avastin, which is an anti-VEGF protein drug. This result indirectly proves the effect of artemisinin in DR: VEGF receptor 2 (VEGFR2) was significantly changed, and several proangiogenic cytokines, including protein kinase C α isoenzyme (PKCα) and platelet-derived growth factor receptor (PDGFR), were also reduced, indicating that the multitarget action of artemisinins may resolve the limitations and adverse reactions of anti-VEGF drugs (Zong et al., 2016). Moreover, Dong et al. also found that endothelial cell proliferation and migration were inhibited by dihydroartemisinin, which targets VEGFR2, which is realized by blocking NF-κB signaling (Dong et al., 2014). Additionally, suppressing ERK1/2 expression and its downstream effectors c-fos and c-myc may be another pathway by which dihydroartemisinin inhibits HUVEC proliferation (Dong et al., 2015). Cheng et al. observed that artesunate therapy induced reactive oxygen species (ROS) generation, and these ROS promote the apoptosis of HUVECs by activating p38 MAPK (Cheng et al., 2013). Extensive evidence indicates that diabetes can cause oxidative stress in the retina and capillary cells, and the increase in reactive oxygen species also damages the structure and function of mitochondria (Duraisamy et al., 2018; Volpe et al., 2018; Wu et al., 2018a).

In terms of antioxidant stress, Yan et al. also demonstrated that artemisinin can prevent retinal pigment epithelial cells growing in high-glucose from oxidative stress via the MAPK/CREB pathway (Yan et al., 2017). Chong and Zheng demonstrated that artemisinin was able to suppress H_2_O_2_-induced oxidative stress in D407 retinal pigment epithelial cells, which are first damaged in retinal diseases through the activation of ERK/cAMP-response element-binding protein (CREB) signaling (Chong and Zheng, 2016). In Li’s research, artemisinin protection of H_2_O_2_-induced human retinal pigmented (D407) cells relied on decreased ROS generation via the activation of AMPK. In addition, artemisinin recovered mitochondrial function, restoring the mitochondrial membrane potential that had been decreased by H_2_O_2_ (Li et al., 2019b). These results illustrated that artemisinin induces the generation of ROS to promote endothelial cell apoptosis in diabetic proliferative retinal diseases, but under oxidative stress conditions, when reactive oxygen species accumulate, artemisinins can protect cells from damage. Therefore, we speculate that artemisinins act as a “balancer,” playing a bidirectional regulatory role in the treatment of Dr. Kowluru et al. suggested that the regulation of mitochondrial homeostasis through antioxidants may provide a treatment modality for the treatment of diabetic retinopathy (Kowluru et al., 2015). Both direct and indirect evidence suggests that artemisinins have great potential in the treatment of DR as antiangiogenic drugs, balancers of oxidative stress, and regulators of mitochondrial function.

### Potential Benefits of Treating Diabetic Cardiovascular Disease With Artemisinins

Because of its high mortality, diabetic cardiovascular disease is one of the most concerning diabetic complications, including macrovascular disease, which mainly involves the coronary artery and aorta, and microvascular disease, which involves diabetic cardiomyopathy (Balakumar et al., 2016; Henning, 2018). Metformin, as a first-line medication for diabetic patients, also shows benefits, including a reduction in cardiovascular events (Maruthur et al., 2016). Cardiovascular benefits are among the most important standards for evaluating diabetes drugs in the clinic.

Diabetic cardiomyopathy (DCM) is an important factor affecting the survival rate of diabetic patients (Kannel et al., 1974; Nichols et al., 2004). The early pathological manifestations of DCM are inflammation and fibrosis of cardiomyocytes, followed by apoptosis and necrosis of cardiomyocytes. Li et al. surprisingly found that artemisinin not only relieved symptoms of T2DM, such as polydipsia, polyphagia, and polyuria, but also ameliorated the general states of DCM in rats. Specifically, it lowered plasma glucose levels and improved cardiac function, such as the left ventricular end-systolic dimension, left ventricular end-diastolic dimension, and left ventricular ejection fraction, by inhibiting high glucose-induced early inflammatory responses, especially by decreasing TNF-α and NF-κB levels, reducing the deposition of collagen fibers and inhibiting myocardial fibrosis in terms of the downregulated expression of TGFβ-1, Collagen Ⅰ, and Collagen Ⅲ (Li et al., 2016).

Abundant evidence shows that after artemisinin therapy, the area of aortic root lesions shrunk, vascular smooth muscle cell hyperplasia and fibrosis were attenuated, and the progression of atherosclerosis lesion formation was diminished, indicating the potential therapeutic effects on atherosclerosis, which is one of the most common manifestations of diabetic cardiovascular disease. Thus, we deduced that artemisinins may alleviate diabetic cardiovascular disease by inhibiting the occurrence and development of atherosclerosis (Wang et al., 2013; Jiang et al., 2016; Du et al., 2019; Cao et al., 2020; Jiang et al., 2020).

Atherosclerosis is a chronic inflammatory disease, and macrophages are the main immune cells involved in atherosclerotic inflammation (Bories and Leitinger, 2017; Moore et al., 2018). In protecting the cardiovascular process, AMPK seems to be the target for artemisinin to reverse pathological changes in activated macrophages. On the one hand, artemisinin significantly promotes phosphorylation of AMPK, followed by suppressing the NF-κB pathway (Cacicedo et al., 2004; Okayasu et al., 2008). Subsequently, the NF-κB network is considered to be the basis of NLRP3 inflammasome activation, which induces the generation of the inflammatory cytokines IL-1β and IL-18 (Bauernfeind et al., 2009). The artemisinin and artesunate groups showed not only an almost complete reversal of elevated NLRP3 inflammasome-related protein expression but also the downregulation of the proinflammatory cytokines TNF-α and IL-6 and the inflammatory chemokines IL-8 and MCP-1 in phorbol 12-myristate 13-acetate (PMA)-induced human monocytic THP-1 cells (Wang et al., 2011a; Jiang et al., 2016; Wang et al., 2016b). Moreover, artemisinin can induce AMPK phosphorylation and suppress NF-κB translocation, inhibiting the expression of VCAM-1 and ICAM-1, which are considered the main adhesion mediators, blocking monocyte-endothelial cell interactions and attachment (Ouchi et al., 2000; Wang et al., 2016b; Jiang et al., 2020). Similarly, the mitogen-activated protein kinase (MAPK) signaling pathway, which is downstream of the NF-κB signal transduction pathway in TNF-α-stimulated HUVECs, also participates in the pathogenesis of these features (Wang et al., 2016b). In the artemisinin group, the expression of phosphorylated ERK1/2, p38, and JNK downregulated and EMMPRIN and MMP-9 activity were decreased (Wang et al., 2011a; Lee et al., 2014; Cao et al., 2015).

On the other hand, a deficiency of macrophage autophagy accelerates foamy macrophage transformation (Shao et al., 2016). Treating HFD-fed ApoE-/-mice with 50, 100 mg/kg/d artemisinin for 8 weeks successfully attenuated foamy macrophage transformation and enhanced macrophage autophagy (Cao et al., 2020). Similar results were obtained in the experiments performed *in vitro*. Mammalian target of rapamycin (mTOR) and uncoordinated-51-like kinase 1 (ULK1) phosphorylation was inhibited upon artemisinin administration, and some autophagic markers were increased, such as LC-3II, which accumulated, and P62 was degraded, showing the enhancement of macrophage autophagy in the oxLDL-treated mouse macrophage cell line (Cao et al., 2020). Furthermore, inflammation disturbs the normal function of vascular endothelial cells and smooth muscle cells (Reglero-Real et al., 2016; Li et al., 2018). Artemisinin decreases the expression of proliferating cell nuclear antigen (PCNA) and the proliferation and migration of rat vascular smooth muscle cells (VSMCs) (Cao et al., 2015). Du et al. provided *in vivo* and *in vitro* evidence demonstrating that artemisinin can decrease PDGF-activated MOVAS migration and proliferation and elevate the expression of contractile phenotypic markers (αSMA, SM22 α, calponin 1, and SMMHC), partly by inhibiting the phenotype switching that leads to a dedifferentiated phenotype (Du et al., 2019). Wang’s team first found that artesunate can significantly increase the expression of KLF2 protein, which regulates the expression of multiple endothelial vascular protection genes (Wang et al., 2013).

Overall, many studies have provided evidence that artemisinins have definitive benefits on the cardiovascular system, but the effects of artemisinin on diabetes-related cardiovascular diseases are still in the preliminary stage. Thus, we still need more evidence to confirm the role of artemisinins in diabetic cardiovascular disorders.

## Artemisinin-Related Side Effects

The limited evidence of drug resistance and low toxicity of artemisinin has been recognized by researchers, whereas the side effects of artemisinin have gradually been recognized with the continuous in-depth study of artemisinin in recent years. As we mentioned earlier, the effects of artemisinin and dihydroartemisinin on INS-1 cell viability gradually increased with increasing concentration, indicating that the toxicity of the two drugs to cells gradually increased with increasing concentration (Chen et al., 2020). Notably, Efferth et al. claimed that protein alkylation is the cause of artesunate-induced toxicity (Efferth and Kaina, 2010). Sun and Zhou also reported that long-term and low-dose exposure to artemisinin might induce free-radical scavengers such as the antioxidant enzyme SOD (Sun and Zhou, 2017), which can destroy the fragile internal peroxide bridge structure in artemisinin, resulting in artemisinin’s reduced treatment efficiency and a dilemma similar to the unrestricted use of antibiotics. Importantly, because of the wide spectrum and nonspecific features of artemisinin, neurotoxicity (Li and Hickman, 2011; Li et al., 2019c), reproductive toxicity (Farombi et al., 2015; Luo et al., 2018), genotoxicity (Singh et al., 2015), etc., have been gradually reported. For example, Singh et al. reported that some unexpected metabolic dysfunctions or abnormalities, including genotoxicity due to sperm DNA damage, might emerge upon excessive artemisinin use. Considering the broad application prospects of artemisinin and its derivatives, we presume that different artemisinin concentrations may be suitable for different pathological conditions. Once the concentration is excessively high or low, artemisinin treatments may cause a number of side effects. Looking for the proper concentration of artemisinin analogs that are suitable in the pathological state of interest may be the next step to recognize the maximum potential of artemisinin, which will be beneficial to clinical applications and will ultimately achieve the goal of precision medicine.

## Discussion

As a range of drugs with huge potential for the treatment of metabolic diseases, artemisinin and its derivatives play critical roles in the therapy of T2DM as well as its related complications. Firstly, through horizontally comparisons of the properties and tissue distribution of artemisinins, it is helpful for us to understand the therapeutic effects and mechanism of artemisinins on diabetes and its complications and provide directions and ideas for future research. For example, artemisinins, except arteether, can pass through the blood-brain barrier; thus, we can infer from the experimental results of Albasher and Zeng that artemisinin may attenuate diabetic cognitive impairment (Zeng et al., 2017; Albasher et al., 2020). Because of these characteristics, artemether, dihydroartemisinin, and artesunate deserve further study to determine their effects on diabetic cognitive impairment. Moreover, artesunate and artemether are distributed in skeletal muscles and liver, which may align with their function of ameliorating IR. Similarly, artesunate distributed in the eyeball has also been confirmed to alleviate eye diseases (Cheng et al., 2013; Ge et al., 2019). Furthermore, artemisinin can pass through blood-placenta barriers (Niu et al., 1985), and artesunate can be distributed in testicular tissue; therefore, we need to pay attention to the reproductive toxicity of these two drugs. The pharmacological understanding of artemisinin and its derivatives is incomplete, and there are still many gaps that need to be filled (Karbwang et al., 1997; Navaratnam et al., 2000; Gautam et al., 2009).

Attenuating IR and restoring islet cell function are two pathways through which artemisinins can alleviate T2DM. Because of their close relationship, obesity, IR, and inflammation often exert cross-influences on each other and continuously promote the development of diseases. Accumulating evidence confirms that artemisinin and its derivatives can break any step of a vicious cycle by modulating adipose production, differentiation, and consumption and inhibiting the inflammatory response to reverse metabolic dysfunction. Artemisinin and its derivatives also act on islet cells by promoting insulin secretion, protecting pancreatic islet ß cells, and achieving islet α-cell to ß-cell transdifferentiation by reversing a number of abnormal proteins and RNAs, thereby inhibiting the development and progression of T2DM.

In addition to playing a crucial role in treating T2DM, artemisinins can also participate in the therapy of diabetic complications through a series of molecular pathways. Notably, in numerous studies, inhibiting the expression of key inflammatory pathways or inflammatory factors and attenuating the chronic inflammation state will significantly improve the functions of related organs. Attenuating inflammation seems to be an important therapeutic mechanism, even for diabetes and all the complications that have been studied. In addition, artemisinin and its derivatives have demonstrated great promise as regulators of oxidative stress, especially in diabetic nephropathy, diabetic retinopathy, and diabetic cognitive impairment. Specifically, artemisinins ameliorate DKD by regulating metabolism, restoring mitochondrial function, modulating RAS, and altering a range of related abnormal molecules. The technology of next-generation sequencing can be applied to identify related genes and pathways influenced by artemisinin. For DR, according to one study, artesunate is more effective than the anti-VEGF drug Avastin and can also effectively prevent the occurrence of retinal detachment (Zong et al., 2016). On the one hand, artesunate can induce apoptosis of epithelial cells and reduce neovascularization by inducing oxidative stress; on the other hand, artemisinin can protect retinal epithelial cells from damage caused by the oxidative stress state of diabetes (Zong et al., 2016; Yan et al., 2017). Therefore, we speculate that the effects of artemisinin are different based on the different pathological conditions of epithelial cells. Generally, artemisinins act as balancing agents and thus can reverse the pathological state and stabilize the intracellular environment. The protective effects of artemisinins on nerve cells are also significant. In addition to anti-inflammatory and antioxidative effects, artemisinins also decrease the level of Aβ, upregulate neurotrophic factors, modulate the apoptosis of neurons, and change the composition of the gut microbiota to e reverse cognitive impairment. Cardiovascular benefits are among the criteria for evaluating diabetes drugs. In addition to reducing early inflammation, reducing fiber formation can also help prevent the occurrence and development of diabetic cardiomyopathy. It is also worth noting artemisinin’s cardiovascular protective function. Metformin also has a positive role in protecting the cardiovascular system, and artemisinin shows certain advantages that are similar to metformin to some degree. Artemisinins play protective roles in atherosclerosis by inhibiting inflammation, promoting macrophage autophagy, and improving the expression of endothelial vascular protection genes. However, the toxicity of artemisinin and its derivatives has gradually been recognized. The side effects of artemisinin and its derivatives have become an obstacle to the treatment of diabetes. In addition to inducing possible damage to islet ß cells (Chen et al., 2020), artemisinins also have adverse effects upon its inappropriate use in the course of treatment and doses, which limit clinical application and need to be addressed seriously (Farombi et al., 2015; Singh et al., 2015; Cao et al., 2020). Although artemisinins have not shown obvious toxicity in various experiments, ways to reduce side effects to the greatest extent possible while retaining the maximum therapeutic effect may be the next issues to be addressed.

The hypoglycemic effects of artemisinin have been verified in most experiments, but the differences in the experimental results and some questions are still worth further study. 1) Different animal models should be considered. Sex has been considered to play an important role in the metabolism of artemisinin and its semisynthetic derivatives. The free fraction of artemisinin in the plasma of male rats was significantly lower than that of female rats (Ashton et al., 1999). Most studies use a single-sex model, and the effects of sex on the results were not studied, which is a limitation to clinical application. Therefore, it is crucial to perform further studies to assess the general applicability of artemisinin and its derivatives for the treatment of diverse patients with diabetes. The use of different mouse strains may be one of the most likely reasons for the discrepant results. In Ins1-H2B-mCherry × Gcg-Cre × Rosa26-stop-YFP triple transgenic mice and Glucagon-CreERT2; Rosa-LSL-eYFP male mice, α-cell to ß-cell transdifferentiation was not observed; however, they were observed in the αTC1 and Min6 cell lines and transgenic (Gcga: GFP)^ia1^ − (ins: NTR-mcherry)^ml10^ zebrafish. Designing a model that better fits the simulated situation may lead to a conclusion more in line with the true situation. 2) Different mediators should be considered. Different media can cause the same result from different processes; for example, palmitate, high glucose levels, and proinflammatory cytokines can cause ß-cell failure through different pathways. Artemisinin and its derivatives have been shown to respond to reverse inflammatory factor-induced ß cell damage but not to palmitate-induced ß cell damage. It has been demonstrated that artemisinins exert effects in an inflammatory environment rather than in a state where free fatty acids are abundant. Therefore, assessing the disease states of the body in which artemisinin functions may expand the scope for the application of artemisinins. 3) Dose and duration should be considered. Side effects are different based on the dose and duration of artemisinin treatments in different diseases. By reviewing the adverse effects, we found that much attention should be paid to suitable artemisinins, reasonable doses and courses of treatment. Hence, artemisinin analogs can be better exploited for therapeutic interventions. 4) Relatively unknown areas should be explored. Traditional Chinese medicine, such as berberine, is potent in modulating gut microbiota (Zhang et al., 2012; Zheng et al., 2018). Different artemisinin derivatives might have differential roles in changing the gut microbiota. In addition, different dietary and living conditions of the mice may lead to different outcomes through changes in the gut microbiota. 5) Without improved technology, efficacy may be affected by contaminating proteins and RNAs; the original droplet-based single-cell transcriptome contains up to 20% contaminating transcripts, indicating that there are still some defects in the detection and extraction methods at this stage. The development of a greater number of efficient separation and purification methods and techniques will greatly improve the accuracy and the precise understanding of the drug effects. 6) Species-dependent effects need to be considered. Marquina-Sanchez’s report demonstrated that the efficacy of artemisinin is species-dependent. Although abundant evidence shows the great potential of artemisinins in the treatment of T2DM and related complications in diabetic models, there is still reasonable doubt about its efficacy in humans. 7) Multitargeted effects need to be explored. From reviewing various experiments, we suggest that artemisinins are multitargeted in the treatment of diabetes and its related complications. However, there are many unresearched but very valuable targets for the reversion of diabetic pathological changes. Further research will contribute to a comprehensive understanding of the roles of artemisinins in T2DM.

In summary, artemisinin and its derivatives play vital roles in the treatment of T2DM, while the clinical application of artemisinin is still challenging. It is essential to further study the interaction between artemisinins and T2DM and then provide clear reasons to use artemisinin as a potential treatment for T2DM and its complications, paving the way for the future cure of diabetes in patients.

## Author Contributions

RS-Y is the corresponding author of the study. YY-J is the first author and responsible for collecting materials and writing the paper. JC-S helped organizing the information and edited the article pictures. BX-Z and JW-C are responsible for the second check. All authors read and approved the final article.

## Conflict of Interest

The authors declare that the research was conducted in the absence of any commercial or financial relationships that could be construed as a potential conflict of interest.
